# Automated circuit fabrication and direct characterization of carbon nanotube vibrations

**DOI:** 10.1038/ncomms12153

**Published:** 2016-07-11

**Authors:** G. Zeevi, M. Shlafman, T. Tabachnik, Z. Rogachevsky, S. Rechnitz, I. Goldshtein, S. Shlafman, N. Gordon, G. Alchanati, M. Itzhak, Y. Moshe, E. M. Hajaj, H. Nir, Y. Milyutin, T. Y. Izraeli, A. Razin, O. Shtempluck, V. Kotchtakov, Y. E. Yaish

**Affiliations:** 1 Andrew and Erna Viterbi Faculty of Electrical Engineering, Technion, Haifa 32000, Israel

## Abstract

Since their discovery, carbon nanotubes have fascinated many researchers due to their unprecedented properties. However, a major drawback in utilizing carbon nanotubes for practical applications is the difficulty in positioning or growing them at specific locations. Here we present a simple, rapid, non-invasive and scalable technique that enables optical imaging of carbon nanotubes. The carbon nanotube scaffold serves as a seed for nucleation and growth of small size, optically visible nanocrystals. After imaging the molecules can be removed completely, leaving the surface intact, and thus the carbon nanotube electrical and mechanical properties are preserved. The successful and robust optical imaging allowed us to develop a dedicated image processing algorithm through which we are able to demonstrate a fully automated circuit design resulting in field effect transistors and inverters. Moreover, we demonstrate that this imaging method allows not only to locate carbon nanotubes but also, as in the case of suspended ones, to study their dynamic mechanical motion.

## Abstract (web summary)

Integrating carbon nanotubes into electronic devices requires quick and non-invasive imaging of the nanostructures for precision positioning. Here, the authors use the base of the nanotubes to nucleate the growth of optically visible organic nanocrystals, which thus enables simple microscopy.

## Graphical abstract

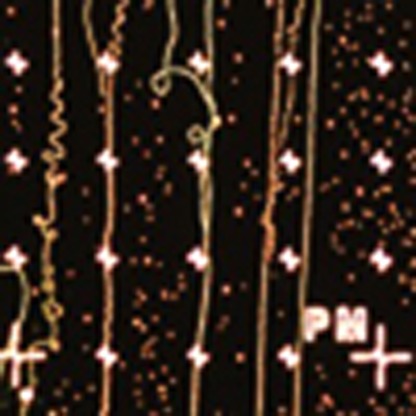

Carbon nanotubes (CNTs) are considered an attractive candidate for future electronics^1^,^2^,^3^,^4^,^5^. Several approaches were developed to harness their unique properties for different applications. Some studies devote their efforts to grow CNTs between predesigned locations^6^,^7^,^8^,^9^ or to accurately deposit them from solution^10^. Alternatively, circuit design can be performed post deposition of the CNTs assuming their position and alignment are known. Currently, the two most common imaging techniques are based on scanning electron microscopy (SEM) or atomic force microscopy (AFM). Both methods are invasive, slow and difficult to scale up.

Alternatively, optical imaging techniques for CNTs are faster, less invasive and provide structural information on the tubes, that is, chirality and type. These methods include Raman spectroscopy, Rayleigh scattering, modulation absorption, polarization absorption, fluorescent spectroscopy and conductance imaging by either photocurrent or photothermal microscopy^11^,^12^,^13^,^14^,^15^,^16^,^17^,^18^,^19^. However, most of these methods are based on weak interaction of polarized laser light with the CNT itself, and as a result, the obtained signals are very small in comparison with the background signals. Improving signal to noise ratio requires either invasive sample preparation, specific substrates, aligned CNTs, metallic contacts or specific CNT type, that is, semiconducting CNTs. It is hard to see how these techniques image dense CNT networks with CNTs that cross each other, or CNTs that are bended. Moreover, the requirement that the polarization of the incident light will be parallel to the tube axis imposes a severe constraint for large scale imaging with these techniques. For example, for a given light polarization, the faster mentioned methods can image an area of 100 × 100 μm^2^ in a few tens of seconds.

Other imaging techniques are based on anchoring different objects to the CNT surface such as fluorescent molecules, metallic nanoparticles and some other adsorbents^20^,^21^,^22^,^23^,^24^. The common denominator of such markers is that they bind strongly to the CNT surface, either through covalent bonds or π stacking, and as a result, it is hard to remove them after imaging, thus, altering or damaging the properties of the pristine tubes.

In almost all practical scenarios the CNTs would be used on an otherwise flat surface. This motivated us to study the potential of the CNT to act as a nucleation centre for the growth of optically visible nanocrystals (NCs). Namely, we dress the CNT with large enough NCs (few tens of nanometres) to make it visible (like pearls on a chain).

In the following, we present rapid and non-invasive dark field optical imaging of CNTs using organic NCs which preferentially nucleate along the CNT sidewalls. After imaging, the NCs completely remove without leaving any residue on the CNTs and their electrical behaviour remains identical to that before adsorbing the NCs. Homemade image processing software uses the optical image and automatically finds the CNT locations and designs the optimal electrical layout according to predefined rules. Examples of p- and n-type field effect transistors (FETs) and inverters are demonstrated. The optical imaging technique is effective for imaging suspended CNTs as well. Herein, we actuate piezoelectrically or electrostatically the mechanical motion of the suspended CNTs and optically observe their vibrational frequencies, both in the linear and non-linear regimes (Duffing oscillator), and reveal transition from hardening to softening behaviour as the chamber pressure decreases. All these results are discussed within the framework of a finite element model (FEM) specifically developed by us for explaining the experimental results.

## Results

### Optical imaging of CNTs

**Figure 1:**
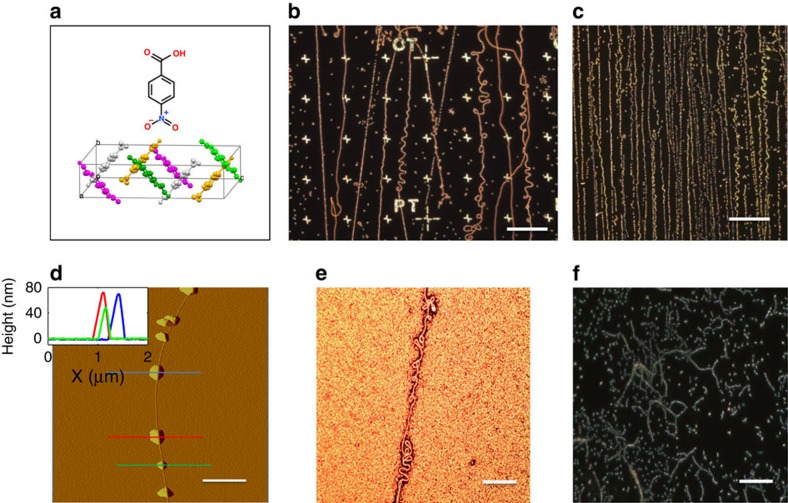
Preferentially adsorption of *p*-nitrobenzoic acid on carbon nanotubes. (**a**) Top: Chemical structure of *p*-nitrobenzoic acid (*p*NBA). Bottom: Schematic illustration of the monoclinic unit cell of *p*NBA powder as extracted from X-ray diffraction analysis. (**b**,**c**) Dark field optical microscopy images of *p*NBA nanocrystals adsorbed along CVD grown carbon nanotubes (CNTs). Scale bar, 50 and 20 μm, respectively. (**d**) Amplitude image of AFM of a single CNT with a few *p*NBA nanocrystals along. Scale bar, 1 μm. Inset: height cross sections along the marked lines of the main figure. (**e**) Dark field optical microscopy image of *p*NBA nanocrystals after intensive deposition. Note the black voids along the CNT. Scale bar, 20 μm. (**f**) Dark field optical microscopy image of *p*NBA nanocrystals adsorb onto commercial dispersed CNTs. Scale bar, 20 μm.

A candidate molecule that could nucleate and grow preferentially over the CNT is *p*-nitrobenzoic acid (*p*NBA) which is drawn schematically at the top part of Fig. 1a. This molecule is small so that it should sublime at relatively low temperatures. The crystal properties of *p*NBA powder were verified using X-ray diffraction (see details in Supplementary Note 1, and Supplementary Fig. 1) and it was found to have a monoclinic unit cell with cell parameters of *a*=2.453 nm, *b*=0.505 nm, *c*=1.291 nm and *β*=93.150002° (bottom part of Fig. 1a). To study the crystal formation on the CNT seed we used several deposition methods which are detailed in Supplementary Note 1. Briefly, in most cases, we first used a silicon wafer covered by thin oxide layer to pattern catalyst pads from which CNTs were grown using chemical vapour deposition (CVD) method^6^,^8^,^25^,^26^. Next, the deposition process of *p*NBA, at 150 °C for 90 s, was carried out on the wafers carrying CNTs. Figure 1b,c present dark field optical images of typical results for two substrates with different densities of CNTs. As shown, the molecules nucleated preferentially along the CNTs, forming an organic NCs chain. The arrangement of the NCs around the CNT is revealed by the AFM image and the corresponding cross section of such NCs along the CNT, Fig. 1d. As Fig. 1d shows, the NCs are formed along the CNT in a pearl chain configuration having few tens of nanometres height and one or two hundreds of nanometres width. The height and linear density along the CNT was found to depend on the sublimation temperature and process time of the *p*NBA deposition. A real-time video that captured the deposition process along the CNTs is found in Supplementary Movie 1. In case of a very long process and overexposure of the wafer the entire surface would be covered by *p*NBA crystals. Figure 1e presents dark-field image of a chip following a deposition process having temperature and duration of 180 °C and 300 s, respectively. In this case the CNT is made visible mainly due to it being a preferential nucleation scaffold, which draws the molecules towards it thus leaving two thin empty lines surrounding the CNT.

The results in Fig. 1 show that while one needs to ensure that all the CNTs are marked with the organic NCs, overexposure should be avoided. This is especially true since we aim not only to statically image the CNT but also to follow its mechanical vibrations. Namely, the minimum height for these NCs that can still be imaged by standard dark field optical microscopy is an important parameter. In this context, it is also important to ensure that the optical imaging does capture all the CNTs. Supplementary Fig. 2a–d compare SEM and optical microscope images (see details in Supplementary Note 2). It is clearly observed that all the CNTs have been detected by the optical method, including when the tubes cross each other, or are in great proximity to each other. In addition, CNTs which appear faint in the SEM image (Supplementary Fig. 2d) are clearly visible in the optical image (Supplementary Fig. 2c). Analysis of the minimum height, *h*_min_, for optical detection of a single NC located along a CNT reveals *h*_min_≤25 nm, and the lateral resolution between two adjacent CNTs is ≈250 nm, as shown in Supplementary Figs 3 and 4, respectively. Since the interaction between the light and the decorated CNT is much stronger than with the bare tube, the required integration time for each image should be much shorter with respect to the other optical techniques. Indeed, we found that the minimum integration time required for the new method is below 1 ms for an area of 120 × 120 μm^2^ (Supplementary Fig. 5), and surprisingly, it is not restricted to SiO_2_ substrates only, but it is applicable to different substrates as well, including high-k dielectrics, insulators and transparent substrates. Several examples are shown in Supplementary Fig. 6.

The last step in testing this nucleation and growth as an imaging tool is to verify that it is not limited to CVD grown CNTs. We obtained commercially available CNTs, dissolved them in organic solvent, and dispersed them on a silicon wafer (see Supplementary Note 1 for more details). The optical image obtained following the *p*NBA deposition process is shown in Fig. 1f, clearly demonstrating the generality of the method.

A key question to address is why the NCs are mainly crystalized along the CNT surface? This question brings us back to non-covalent functionalization of CNT for chemical and biological sensing^21^,^27^. It was suggested that the electronic properties of CNTs coupled with the specific recognition properties of immobilized biomolecule can serve as ideal miniaturized nanosensor. However, covalent immobilization deteriorates the CNT electrical performance, and thus non-covalent methods were developed. Most of these methods were based on aromatic part of the immobilized molecule, which is known to interact with the basal plane of graphite via π stacking^28^. We believe that, here as well, the aromatic part of the *p*NBA interacts with the sidewalls of the CNTs that act as a nucleation seed for the organic crystal growth. After immobilization of *p*NBA molecules on the CNT sidewall, other molecules, which adsorb onto the oxide surface, diffuse (or sublime and readsorb) until they crystalize on the immobilized molecules and ionic crystal growth occurs.

A natural question that arises is the possibility for the existence of similar molecules to *p*NBA, which can decorate the CNT sidewalls as *p*NBA does. These additional molecules can shed light on the adsorbing mechanism of *p*NBA molecules to the CNTs. Three criteria should be fulfilled by the candidate molecules: (i) have low mass weight, (ii) are in a solid phase at ambient conditions and (iii) have a benzene ring. Indeed, we found few additional candidates for marking the CNTs with organic NCs, yet, the quality of the obtained results are reduced in comparison with *p*NBA molecules. Supplementary Figs 8–12 and discussion within Supplementary Note 3 summarize our results.

π stacking is not restricted to CNTs only, but is very relevant to other nanostructures, such as single layer graphene or few layers graphene. Enabling imaging of graphene layers by optical means is of great importance. One common method is based on constructive interference of the reflected rays collected from single layer graphene or few layers graphene on top of insulating materials^29^. However, this method is restricted to specific insulating thicknesses, which are not always optimal with device performance. Moreover, fabrication of heterostructures based on graphene and hexagonal boron nitride imposes many challenges due to the lack of optical imaging of these new structures on different insulating layers^30^. Therefore, we tried to mark graphene samples with *p*NBA NCs and make them optically visible no matter on which substrates they lie. Successful results of such deposition and marking procedure are summarized in Supplementary Note 4 and Supplementary Figs 13–17. Briefly, *p*NBA NCs preferentially adsorbed on single and few layers exfoliated and CVD graphene, as well as graphene nanoribbons, on top of different insulating thicknesses, which do and do not support constructive interference according to Blake *et al*^29^.

### Non-invasive imaging

**Figure 2:**
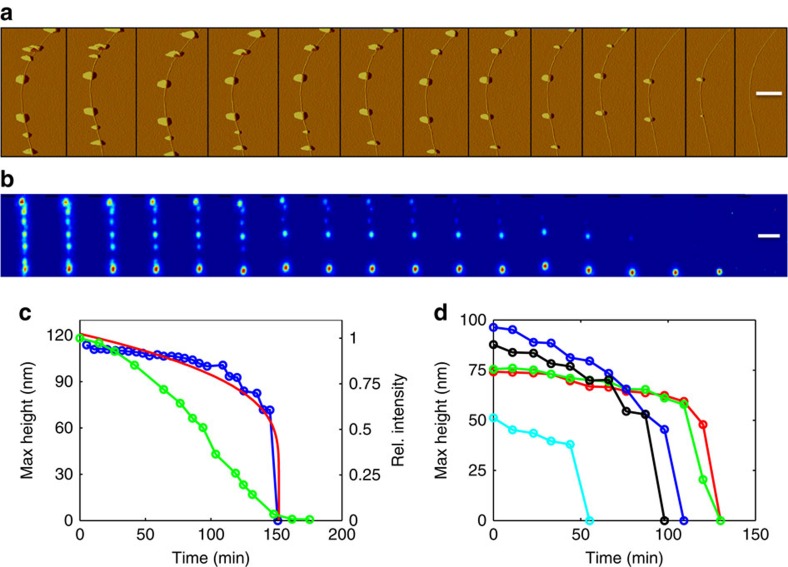
Sublimation rate of *p*-nitrobenzoic acid (*p*NBA) nanocrystals off carbon nanotubes. (**a**) A temporal set of AFM amplitude images of *p*NBA nanocrystals (NCs) along a single carbon nanotube (CNT). The time interval between each image is 11 min. Scale bar, 1 μm. (**b**) A temporal set of dark field optical microscopy intensity of *p*NBA NCs along a single CNT. The time interval between each image is 6.5 min. Scale bar is 2 *μ*m. (**c**) Left axis: NC height as function of time (blue circles) and theoretical fit according to Supplementary Eq. 7 (red line). Right axis: Relative dark field optical microscopy intensity of *p*NBA NC along CNT as a function of time (green circles). (**d**) Temporal dependence of the maximum height of different NCs along different CNTs.

Having established the new mechanism, of nucleation and growth, as an efficient marking method one should verify that following such a step the CNTs maintain their electrical and mechanical properties. The first indication would be if the *p*NBA NCs would completely desorb off the CNTs leaving no residue behind. Figure 2 describes the sublimation process under ambient conditions for these *p*NBA NCs, that is, after they were first grown and nucleated along the CNTs. Figure 2a,b depict the temporal dependence of the AFM and optical images of two marked CNTs with similar *p*NBA NCs. We note in Fig. 2b that the CNTs remain optically visible for about an hour, where, Fig. 2a shows that the complete desorption would require an additional hour. As in any desorption study, the process can be accelerated by elevating the temperature or by introducing the sample into vacuum. To ensure that the sublimation process off the CNT follows a classical model such that full desorption could be predicted we present in Fig. 2c the temporal evolution of the NCs size as represented by the maximum height, *h*_NC_ (left axis, blue circles), and the relative optical intensity (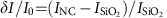; right axis, green circles). The red line in Fig. 2c is best fit to a classical sublimation model and the detailed analysis is presented in Supplementary Note 5. Height evolution for a range of marked CNTs is depicted in Fig. 2d. One can notice that the general process is similar, starting with a linear decrease until a critical height is obtained where the process accelerates. In fact, it is quite striking that although the deposited NCs were enormously large in comparison with the pristine CNT diameter, the final height shows no indication for the presence of *p*NBA molecules. To support this conclusions transmission electron microscopy (TEM) imaging was conducted (see Supplementary Note 2 for more details). Briefly, CNTs were grown on TEM grids and imaged before intensive *p*NBA molecules deposition and after they sublimed off the CNT. Supplementary Fig. 7a–d presents the results of this analysis, and confirm that no residues are left on the CNT surface indicating that complete desorption is achieved. Supplementary Fig. 7e,f shows typical optical images of the decorated TEM grid immediately after *p*NBA deposition, ensuring that indeed all the CNTs within the holes were marked with the NCs.

**Figure 3:**
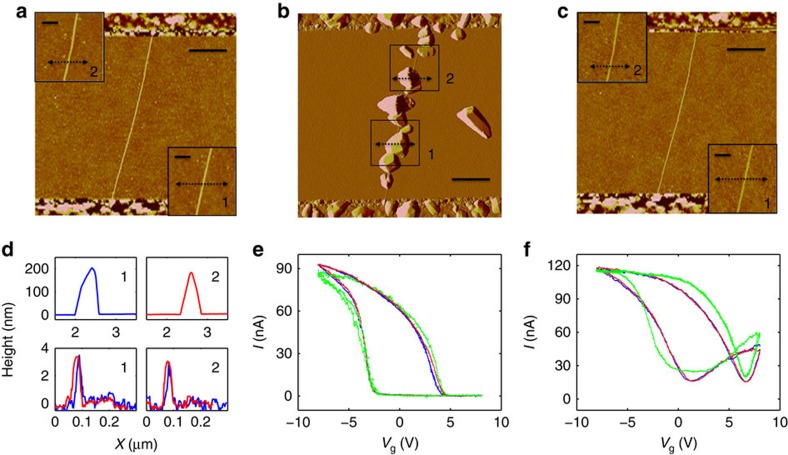
Electrical characterization of marked and non-marked carbon nanotubes. (**a**–**c**) AFM images of complete carbon nanotube (CNT) device with metallic source and drain electrodes, before *p*-nitrobenzoic acid (*p*NBA) deposition (**a**) immediately after (**b**) and after one day (**c**). Zoom in on the black square areas marked 1 and 2 of the main panel of **b** are shown as four insets before deposition (**a**) and after one day (**c**). The dotted lines mark the locations of the cross sections which are plotted in **d**. The scale bar of the main panels, (**a**–**c**) is 1 μm, and for the insets is 200 nm. (**d**) Cross sections along the black dotted lines of panels (**a**–**c**) of area 1 (left) and area 2 (right). Top panels depict the nanocrystals (NCs) height immediately after deposition (blue for area 1, and red for area 2). Bottom panels plot the CNT diameter before (blue) and long after (red) within area 1 (left) and area 2 (right). (**e**,**f**) Transfer characteristic of two CNT devices before deposition (blue), immediately after deposition along the CNT (green), and after cleaning process (red).

To complete the analysis and verify that not only do the NCs desorb but also that the CNT properties remain intact we repeated the procedure on CNT field effect transistors (CNTFETs) where the CNT are contacted by metallic electrodes on both sides. Figure 3a–d presents AFM images and the corresponding cross sections before *p*NBA deposition, immediately after and after sufficiently long time (≈1 day). The electrodes can be seen at the top and bottom of the sub figures. Two black square areas marked 1 and 2 in the main panel of Fig. 3b depict 1 μm^2^ areas where we zoomed in before and after *p*NBA deposition. The zoomed images are presented as insets to Fig. 3a,c and are marked with 1 and 2 according to Fig. 3b. The cross sections of these images were taken along the dotted black lines and are depicted in Fig. 3d. At the top part of Fig. 3d, the cross sections of area 1 (left, blue) and area 2 (right, red) immediately after deposition are plotted. It is evident that the height of these two NCs is above 150 nm. At the bottom of Fig. 3d, the cross sections of area 1\2 (left\right) before (blue) and sufficiently long time after (red) deposition are plotted. The cross sections look similar, and the average height before and after depositions are practically identical (*h*_CNT_=3.25±0.25 nm). These results are in agreement with those reported in Fig. 2 indicating that the presence of the electrodes and the transistor fabrication process do not affect the desorption process. The critical test of the electrical performance of the CNTFET at the stages corresponding to Fig. 3a–c is shown in Fig. 3e,f for two different devices. Figure 3e,f depicts typical transfer characteristic of semiconducting (Fig. 3e) and small band gap (Fig. 3f) CNTFETs before molecule adsorption (blue), immediately after (green), and long after complete desorption (red). Interestingly, immediately after deposition a small shift in the threshold voltage is found, but after cleaning through the desorption process, the electrical response returns to its original values and is identical to the one measured before deposition of the *p*NBA NCs. These results were confirmed using many CNTFETs (above 20) so that we can safely conclude that indeed the process of marking the CNTs, which includes adsorbing of *p*NBA on the CNT surface, organic crystal growth and later on, complete desorption, does not leave any residue, and does not affect the overall electrical performance of the complete device.

**Figure 4:**
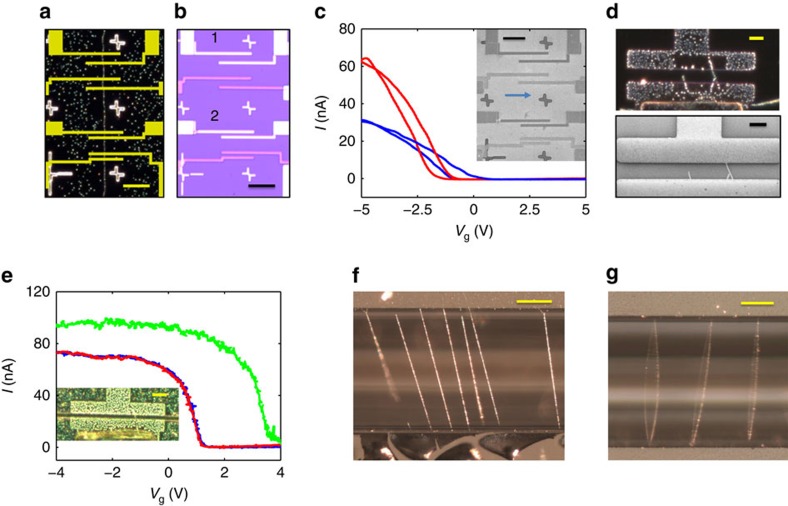
Optically imaging of on-surface and suspended carbon nanotube devices. (**a**) Dark-field optical microscopy image of marked carbon nanotube (CNT) with *p*-nitrobenzoic acid (*p*NBA) nanocrystals (NCs), and overlay of the lithography circuit design. Scale bar is 10 μm. (**b**) Optical image of the complete fabricated device. The two different colour electrodes are two different metallic contacts. Scale bar is 10 μm. (**c**) Transfer characteristic of the two CNTs devices 1 (blue), and 2 (red). Inset: SEM image of the completed circuit. Blue arrow points towards the CNT that was marked in (**a**). Scale bar, 10 μm. (**d**) Top: Dark field optical microscopy image of marked junction. Bottom: SEM image of the same junction. Scale bar in both images is 3 μm. (**e**) Main panel: Transfer characteristic of suspended CNT before *p*NBA deposition (blue), immediately after deposition (green), and after cleaning process (red). Inset: Dark field optical microscopy image of the measured junction marked with *p*NBA NCs. Scale bar, 3 μm (**f**,**g**) Dark field optical images of long suspended CNTs decorated with *p*NBA NCs. Scale bar, 50 μm.

On the basis of the above results one can envisage CNT circuit fabrication procedure that consists of growing the CNTs, marking the CNTs on the given substrate with *p*NBA molecules, acquiring the image using an optical microscope, and designing the electrical circuit accordingly. Figure 4a presents an overlay of the microscope image of marked CNT with the electrical circuit design that was made accordingly. The optical image of the completed circuit, as fabricated, is shown in Fig. 4b and its SEM image is shown in the inset of Fig. 4c. The different colours of the source and drain electrodes, Fig. 4b, are attributed to two different metals used for the electrodes (Gold and Aluminium). The blue arrow, Fig. 4c inset, points to a faint line that corresponds to the CNT, see also Fig. 4a. Figure 4c shows the measured transfer characteristic of devices 1 (blue line), and 2 (red line), as depicted in Fig. 4b.

This result confirms that indeed the method presented here is successful and efficient for the fabrication of CNT based devices. We have used this method and fabricated more than 100 CNT-based devices, with more than 95% success rate (see details in Supplementary Note 6). The high yield is attributed to the exact location of the complete CNT network and the possibility to place our electrodes at optimal locations, where the tubes are straight, far from other tubes and do not split to additional tubes. Supplementary Figure 18a, b depicts two histograms for the total resistance and resistance per micron length of the fabricated CNT devices produced by the described method. These results are extremely good and are well within the high-end group of existing devices.

To contrast our method with the case where the CNTs position is not known, and hence is not used as an input to the electrode design, we blindly fabricated devices on CNTs network. The marking by *p*NBA and optical imaging were carried out only after the devices were completed and the images are shown in Fig. 4d,e. Surprisingly, the images show combinations of CNTs bridging narrow junctions as small as 1 μm and suspended tube. The top part of Fig. 4d shows a dark-field optical image of a 3 μm gap junction and the bottom part shows a SEM image of the same junction. Comparing the two images, it is clear that the *p*NBA marking procedure accurately stains all the CNTs within the electrode area and that luckily only a single tube is bridging the two electrodes. To show that, as far as the imaging is concerned, post-imaging is robust we present in Fig. 3e the transfer characteristics of this specific junction before, immediately after, and long period of time after *p*NBA deposition. As before, the electrical characteristics fully recover once the *p*NBA is given time to fully desorb. These results suggest that such imaging procedures could not only be used for circuit design but also in failure analysis of CNT circuits thus enabling the full fabrication chain.

### Mechanical vibrations of suspended CNTs

Until now we have mainly discussed the marking procedure of CNTs on surfaces. However, suspended CNTs play a major role in the research and applications of nano-electromechanical systems (NEMS). Due to their high Young's modulus, low mass and presumably low defect density, one can anticipate to obtain extremely high resonance frequencies with high-quality factors^31^,^32^,^33^,^34^. The fabrication challenges for such applications are somewhat similar and with respect to imaging and ensuring a given number of tubes in the gap the challenges are almost identical. Currently, identifying the number of tubes bridging the junction is performed either by SEM or by AFM. SEM usually deteriorates the mechanical properties of the CNT resonator, and shifts their resonance frequencies due to deposited materials while imaging. AFM besides being extremely slow, is very detrimental and often ends with device deterioration or even disconnection. Clearly, non-invasive and rapid imaging procedure is highly required here as well. To demonstrate the feasibility of our method, we have fabricated suspended devices and deposited *p*NBA molecules as described before. The results are presented in Fig. 4e–g. The inset of Fig. 4e shows that for narrow trenches, as small as 1 μm, a clear image of the suspended CNT bridging the two metallic contacts is possible. For narrower trenches, the strong light scattering by the metallic electrodes may obscure the *p*NBA NCs along the suspended tube. In such circumstances, the tube can still be identified through its portions that extend beyond the electrode area and one could even extrapolate the location of the suspended part of the tube from its marked portion on the surface.

As before, the validity of the imaging method is tested by comparing the electrical properties of the CNTs at different stages of the process. The main panel of Fig. 4e presents the transfer characteristic of a suspended CNT, similar to that seen in the inset of Fig. 4e, before (blue), immediately after (green) and long time after (red) deposition of *p*NBA molecules. It is clear that although immediately after deposition the electrical measurements are significantly different, due to the presence of *p*NBA NCs, the two measurements that were conducted before and long time after deposition, coincide completely with each other, as was found previously for on-surface CNTs. Working with suspended CNTs and especially with longer ones it may be possible to image their mechanical motion and examine their dynamics in terms of vibrational modes. For that purpose, we fabricated deep trenches, 80 μm depth, 200 μm wide and grew long and straight CNTs across. Later, *p*NBA molecules were deposited and dark-field optical images of the suspended CNTs were collected. One typical image is shown in Fig. 4f. The suspended tubes are decorated with *p*NBA NCs and are clearly seen by the optical image. SEM image of similar NCs is shown in Supplementary Fig. 2e. They crystalize on the CNT surface and after a short period in high vacuum (required for SEM operation) they form discontinuous segments along the tube. Applying mechanical excitation to long suspended CNTs would typically cause such tubes to oscillate. Figure 4g depicts such dynamical behaviour, where the mechanical excitation was provided by gentle air flow. A real-time video of such oscillations is presented in Supplementary Movie 2. From these images and others, one can optically visualize the vibrational modes of CNTs, including higher modes beside the fundamental one, and obtain important information about their slack, dynamics, non-linear behaviour, temporal and spatial correlations, as well as dissipation processes.

**Figure 5:**
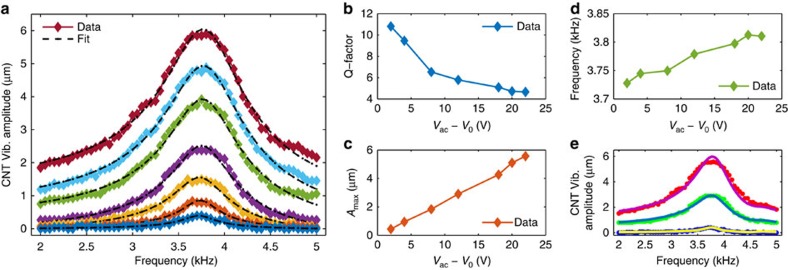
Optically measured mechanical resonances of marked carbon nanotubes. (**a**) Amplitude of vibration of carbon nanotube (CNT) versus excitation frequency of a driven piezoelectric actuator, *V*_ac_. The different coloured curves are for different applied voltages of the piezo as plotted in **c** (2, 4, 8, 12, 18, 20 and 22 V from bottom to top). The different black dashed lines are best fit to Lorentzian curves. (**b**) Quality factors as extracted from the Lorentzian fit. (**c**) Maximum amplitude versus the applied ac piezo voltages (subtracted offset bias originated from the electrical setup, *V*_0_). (**d**) Frequencies of the maximal response for different excitation voltages. The chronological time of the measurements were from right to left. (**e**) The same data as in **a** for three different *V*_ac_'s (dotted coloured lines) and resulted fit (colored lines) according to our finite element model (FEM) discussed in Supplementary Note 7. Vib., Vibration.

Two types of quantitative vibrational experiments were performed, one utilized piezoelectric actuation and the second electrostatic actuation. For both methods, the vibrational modes were detected optically (Supplementary Note 7 and Supplementary Fig. 19). As before, the devices comprised suspended CNTs of different lengths (*L*≈30, 100 and 200 μm) with various levels of *p*NBA NCs decoration along their sidewalls. Figure 5a depicts the frequency response of such CNT for different excitations of the piezoelectric actuator. The data nicely fit to Lorentzian with a single peak, as expected for suspended doubly clamped beam. For the entire excitation range, the behaviour is linear and the quality factors and peak heights are plotted in Fig. 5b,c. However, for different deposition condition, the frequency response contains more than a single peak, as presented in Supplementary Fig. 20a. Naively, for CNTs without slack, *s*, and without built-in tension, *T*_0_, the first vibrational mode is given by solving Euler-Bernoulli equation^35^ to yield 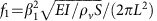 where *β*_1_=1.8751, *E* is the beam Young's modulus, *I* is the moment of inertia, *ρ*_*v*_ is the mass density, and *S* is the beam cross section area. However, when the tube is decorated with the *p*NBA NCs two opposite effects modify the resonance frequencies. The first effect, which scales as *r*^2^, where *r* is the radius of the organic circumference, originates from the increase of the tube total mass, and hence causes reduction of the resonance frequencies. The second effect originates from the bending rigidity attributed to the *p*NBA NCs, which involves their Young's modulus, *E*_*p*NBA_ as well as their moment of inertia, *I*_*p*NBA_, which scales as *r*^4^. For homogenous coverage the effective bending rigidity is given by the core shell model^36^


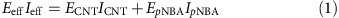


where *E*_CNT_≈1TPa, *I*_CNT_≈(1 nm)^4^, *E*_*p*NBA_≈500 MPa, and *I*_*p*NBA_≈r^4^. Although *E*_*p*NBA_<<*E*_CNT_, for outer radii which are significantly larger than the typical pristine tube radius, the bending rigidity of the shell will play the dominant role in affecting the resonance frequencies. As the shell becomes wider, the total mass of the tube increases as well. However, the mass per length increases as *r*^2^, where the bending rigidity increases as *r*^4^, hence, overall the resonance frequencies will increase in comparison to a bare tube with the same length, as can be observed in Fig. 5a and Supplementary Fig. 20a. Moreover, while measuring the vibrational modes, *p*NBA molecules sublime and leave the CNT, and the resonance modes are expected to decrease, as is observed in Fig. 5d.

In real experiments, the *p*NBA NCs decorate the CNT sidewalls unevenly, thus, a more rigorous analysis is required. For that purpose, we developed a homemade FEM based on numerical simulation that takes into account non homogenous coverage of the CNT sidewalls with *p*NBA NCs (see details in Supplementary Note 7, and Supplementary Fig. 25). The algorithm includes slack as well, which is typically introduced as a consequence of thermal contraction of the fabricated trench on cooling from the hot CNT growth temperature (∼900 °C) to room temperature. Slack plays an important role in affecting the tube resonance modes, dissipation processes^37^, and lifting the degeneracy of the in-plane and out of plane resonance frequencies^38^. Usually, this parameter is deduced from three-point bending experiments^39^, or as a fitting parameter for the obtained vibrational data. Here, thanks to the optical imaging techniques we could directly measure the slack of our fabricated devices, and use it in our analysis for the vibrational modes. Supplementary Fig. 21a shows how we measure the slack of specific CNT by DF microscopy, and Supplementary Fig. 21b presents the distribution of slack in our devices. Knowing the slack enables us to fit the measured vibrational modes to the theoretical predictions according to our FEM. Two successful examples for two different CNTs are presented in Fig. 5e and Supplementary Fig. 20c.

### Optically observed Duffing oscillator of suspended CNTs

**Figure 6:**
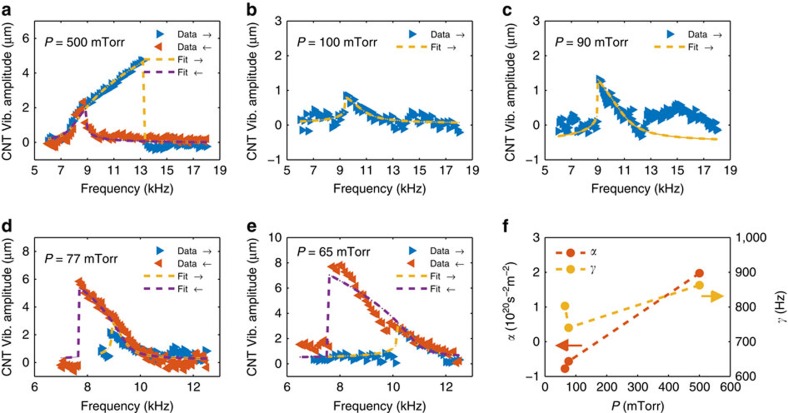
Hardening to softening behaviour. (**a**–**e**) Amplitude of vibration of carbon nanotube (CNT) versus excitation frequency for different pressures, as marked inside each panel. Blue triangles are data for up sweep and red triangles for down sweep. The yellow and purple dashed lines are the theoretical solutions according to Supplementary Equations 33 and 34, for up and down sweep, respectively. (**f**) Left axis: Extracted non-linear spring constant, α, as function of pressure. Right axis: Extracted coefficient of linear damping, γ, versus chamber pressure. Vib., Vibration.

All the previous measurements were preformed at ambient conditions. However, it is also possible to obtain the frequency response also in vacuum with the same optical detection technique. This time, the actuation was based on electric force applied between the tube electrodes and an external probe located near the suspended tube. Surprisingly, for this set of measurements strong non-linear behaviour and hysteresis were observed. Supplementary Fig. 22 shows the vibrational behaviour for different excitation powers. Supplementary Fig. 23 presents the frequency response for different pressures. Figure 6 depicts transition from hardening to softening behaviour as the chamber pressure decreases. These set of phenomena can be understood within the framework of Duffing oscillator^40^. In such lumped model the beam displacement, *u*, satisfies the following differential equation


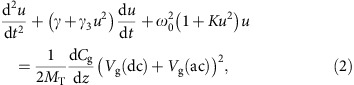


where *K* is responsible for hardening/softening and *γ*_3_ for non-linear damping^41^, *M*_T_ is the total mass of the oscillator, *C*_g_ is the tube-external gate mutual capacitance, and *V*_g_(dc/ac) is the dc/ac external gate voltage applied between the CNT and the metallic probe. Under external dc bias the tube is being stretched and the fundamental resonance frequency increases accordingly. Supplementary Fig. 24a presents such increase under the applied gate voltage and our FEM agrees very well with the experimental data. It should be noted that several *C*_g_ were tested, all described in Supplementary Note 7 and Supplementary Fig. 26, but the resulted fitting parameters are very similar (within 10%). Then, using these parameters, we could fit the Duffing type behaviour, as presented in Supplementary Figs 22, 23 and Fig. 6, according to the rotation frame analysis^40^ discussed in Supplementary Note 7. The model describes very well the experimental data, and even the transition between hardening and softening can be understood from equation 2, as seen in Fig. 6. In that case the force term at the right hand side of equation 2 can be expanded in powers of *u*, to yield terms which are linear and cubic in *u*. The linear term may modify the resonance frequency, where the cubic term may affect the non-linearity term, *K*. As the pressure decreases, and more molecules sublime off the CNT sidewalls, the right hand side becomes more dominant, and the non-linear term is given by


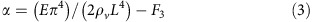


according to Supplementary Equation 31, becomes negative. In such circumstances where the total mass is low, and *V*_g_(dc) is high, α changes sign and the expected hardening behaviour becomes softening, as observed in Fig. 6. Detailed analysis of the non-linear term within the Duffing lumped model (Supplementary Note 7) reveals good agreement between the estimated *α*=−0.45±0.1 10^20^ s^−2^ m^−2^ to the one found at low pressure from the fitted data in Fig. 6d, that is, *α*=−0.56±0.07 10^20^ s^−2^ m^−2^.

### Automatic circuit design and fabrication

As a last example of the power in the method presented here we address the potential of CNTs to compete or integrate with silicon fabrication, an issue considered to be a dream by more than a few. A major milestone for realizing such a task (‘dream') is the high level of device integration used in the silicon industry. Following the design rules of silicon and the idea that all patterns are predetermined before the fabrication process indeed seem impossible in the context of CNTs. Alternatively, one can choose a path where the functionality is indeed fully predetermined but the patterns are designed and fabricated on the fly as part of the process line. On the basis of the results shown above one may suggest that rapid imaging of the CNTs network, together with image processing capabilities and software design tools, will enable automatic fabrication of CNTs based circuits. To prove the feasibility of such a path we developed an image processing software and a pattern design tool that could automatically design the fabrication pattern of simple components.

**Figure 7:**
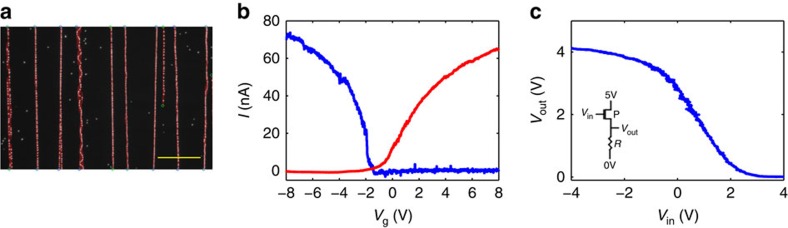
Automatic design and fabrication of carbon nanotubes based devices. (**a**) Image processing results. Dark field optical microscopy image of marked carbon nanotubes (CNTs) (white dotted lines) superimposed with the image processing analysis results (red lines). Scale bar, 30 μm. (**b**) Transfer characteristics of automatically designed p- (blue line) and n- (red line) type CNT field effect transistors (FETs). (**c**) Automatically designed inverter based on p-type CNTFET and external resistor. Inset: schematic diagram for the inverter.

The full fabrication process starts with growing CNTs from pre-patterned catalyst islands on top of silicon substrate with 300 nm SiO_2_. Following the CNTs growth the sample was marked with *p*NBA molecules, and dark-field optical images were collected. The images were fed to our homemade image processing software and the result of finding all the CNTs and their layout is depicted in Fig. 7a. The red lines are the software output which follow exactly the marked CNTs. Details of the image processing algorithm can be found in Supplementary Note 8. Next, using only the software locations of the CNTs, p- and n-type CNTFETs, as well as inverter circuits based on this network were automatically designed according to pre-design rules. An example is shown in Supplementary Fig. 27 and the details of the fabrication process for the p- and n-type FETs are discussed in Supplementary Note 8 as well. The electrical characteristics of the complete devices are plotted in Fig. 7b,c. Two types of inverters were utilized. The first (type I) includes two steps of fabrication, one for the p-type behaviour, and the second for n-type. The second type (type II) was based on p-type CNTFETs and regular resistors. The two approaches were successful, and are plotted in Supplementary Fig. 27c (type I) and Fig. 7c (type II). These p- and n-type CNTFETs, and two kinds of inverters which were automatically fabricated are successful examples for this promising approach.

### Summary

In conclusion, we presented a novel and rapid method based on *p*NBA NCs to optically visualize CNTs. The method is based on our finding that CNT surface constitutes a nucleation seed for organic crystal growth that can then be imaged by dark-field optical microscopy. This non-invasive method was found not to leave any residue on the tube sidewall and consequently to have no effect on the CNTs electrical or mechanical performance. The method was demonstrated on both on-surface and suspended devices down to gap size of 1 μm.

Using relatively low level of NCs coverage we were able to not only statically image the CNTs but also to study the dynamic behaviour of suspended CNTs, including oscillations, vibrational modes, non-linear characteristics, and transition from hardening to softening behaviour. The marking and imaging procedure is scalable and enables automatic design and fabrication of electrical circuits based on CNTs. We demonstrated automatic fabrication of both p and n-type CNTFETs as well as inverters and have shown that the CNTs retain their electrical properties throughout the process. We believe that the suggested method can provide a real platform for the integration of nanoelectronics with silicon technology.

## Methods

### Device fabrication and molecule deposition

CNTs were grown using CVD at 900 °C with 0.5/0.5 SLM flow of H_2_/CH_2_. The catalyst particles were deposited from ferritin solution onto predefined catalyst pads. Electrical contacts were deposited either before or after CNTs growth. For p-type devices Cr/Au 5/120 nm or Cr/Pt 5/40 nm were deposited^6^,^8^,^25^,^26^. For n-type devices four different processes were used. The first was based on 50 nm of Al deposition capped by 50 nm of Au^42^. The second approach included Ca/Al 30/120 nm deposition^43^, and the third consisted of Sc 50 nm metal deposition^44^. The last approach, which was found to be the best, included Cr/Pt 5/40 nm electrical contacts and atomic layer deposition (ALD) of HfO_2_ on top of the CNT^45^. The deposition temperature and pressure were 270 °C and 620 mTorr, and the HfO_2_ thickness was ≈30 nm.

Powder CNTs were purchased from SWeNT and CoMoCAT and were dissolved in chloroform. After tip sonication for 20 min in pulse mode, they were dispersed on silicon wafer. *p*NBA powder was purchased from Fluka. The deposition on the CNTs uses a hotplate. A small metallic tray ∼1 cm^2^ in size is filled with the *p*NBA powder and placed near the edge of a 10 cm petri dish. Only this edge is sited on a hotplate, and a glass lid covers the whole petri dish. The hotplate is heated up to 150 °C for 15–30 min until *p*NBA molecules start to cover the lid. Then, a silicon chip with the CNTs is placed on the cold side of the petri dish, and the molecules condense on the chip. Typically, the condensation period lasts for 90 s, but shorter or longer times result with diluted or denser *p*NBA coverage.

### TEM analysis

Commercial TEM grids with thin silicon nitride mesh film 200 nm thick, and 2 μm diameter holes were used. Catalyst solution was deposited on the circumference of the TEM grid and CNTs were grown using CVD tool. Imaging was performed using 120 kV, LaB6 emitter equipped, FEI T12 G2 TEM, before and sufficiently long time after (2 weeks) *p*NBA deposition.

### Electrical measurements

The electrical measurements were done using either DC or AC currents at zero or few Hertz, respectively. The voltage biases were few mV with few hundreds msec time constants.

### Vibrational data

The suspended tubes were actuated by piezoelectric membrane or electrostatic electrode connected to RF generator. The detection performed optically by automatically analysing the vibrational amplitude image for each frequency excitation within the frequency range.

### Data availability

The authors declare that all relevant data are available from the authors on request.

## Additional information

**How to cite this article:** Zeevi, G. *et al.* Automated circuit fabrication and direct characterization of carbon nanotube vibrations. *Nat. Commun.* 7:12153 doi: 10.1038/ncomms12153 (2016).

## Supplementary Material

Supplementary Information Supplementary Figures 1-27, Supplementary Notes 1-8 and Supplementary References

Supplementary Movie 1 Live deposition of pNBA molecules CNTs

Supplementary Movie 2 Vibrating pNBA marked CNTs
