# Supplementary material for: Automated circuit fabrication and direct characterization of carbon nanotube vibrations
Source: Nat Commun. 2016 Jul 11;7:12153. doi: 10.1038/ncomms12153 (PMC4942577; doi:10.1038/ncomms12153)
Supplement: Supplementary Information — Supplementary Figures 1-27, Supplementary Notes 1-8 and Supplementary References [file ncomms12153-s1.pdf]

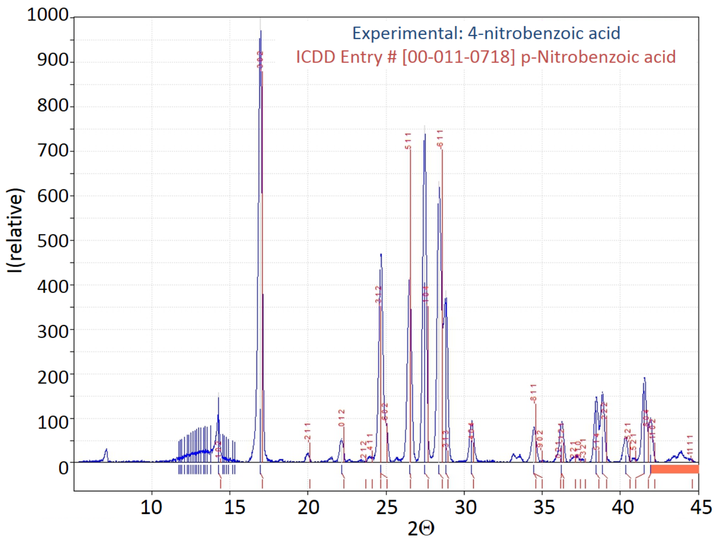

Supplementary Figure 1. **Crystallographic structure**. X ray diffraction pattern of p-nitrobenzoic acid powder with peaks identification (carried out by Match! Software, Copyright 2003-2014 CRYSTAL IMPACT, Bonn, Germany).

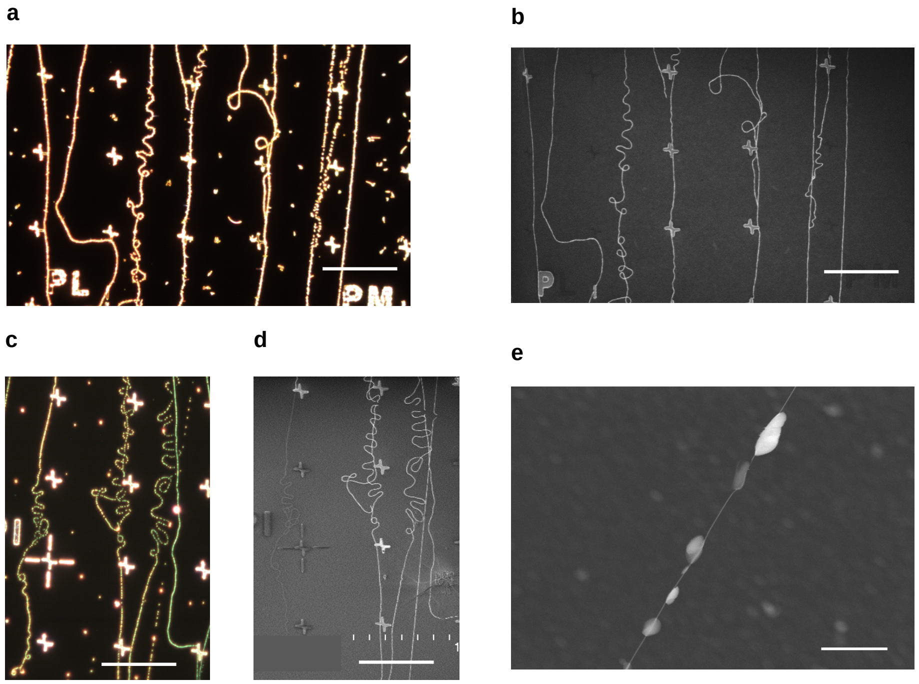

Supplementary Figure 2. **pNBA marked CNT images**. (a-d) Comparison of dark field optical microscopy images (a, c) to SEM images (b, d) of the same area. Scale bar for a-d is 50 μm. (e) SEM image of pNBA NCs along suspended CNT. Scale bar is 1 μm.

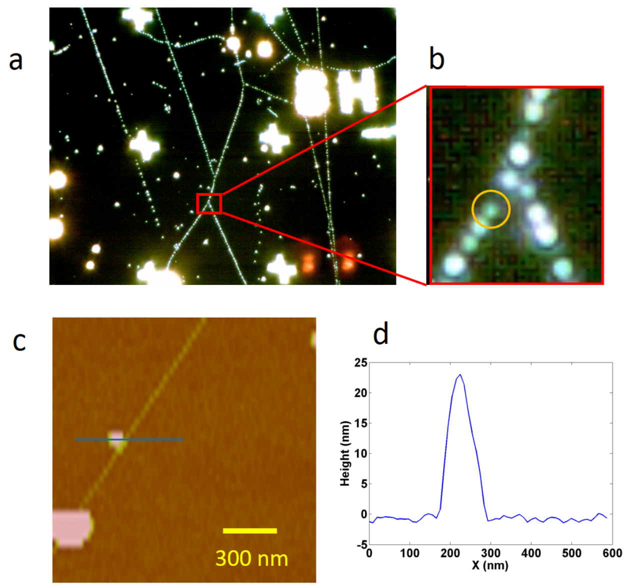

Supplementary Figure 3. Vertical image resolution. Minimum size for pNBA NCs which can be imaged by dark field optical microscopy. (a) Dark field optical image. (b) Zoom in on the red square of a. The yellow circle marks the optically visible pNBA NC on which AFM scan was performed. (c) AFM image of the small NC, and (d) cross section along the blue line of (c).

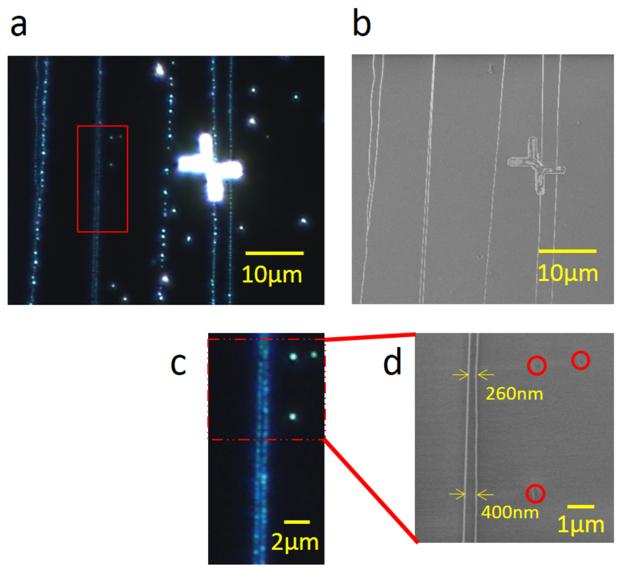

Supplementary Figure 4. Lateral image resolution. Minimum separation length between two distinct CNTs imaged by dark field optical microscopy. (a and c) Dark field optical images. Image c is an enlarge image of the red rectangular area of image a. (b and d) SEM images. Image d is a SEM image of the same area marked by the dashed red square in c. The three red circles in d correspond to the three bright spots in c.

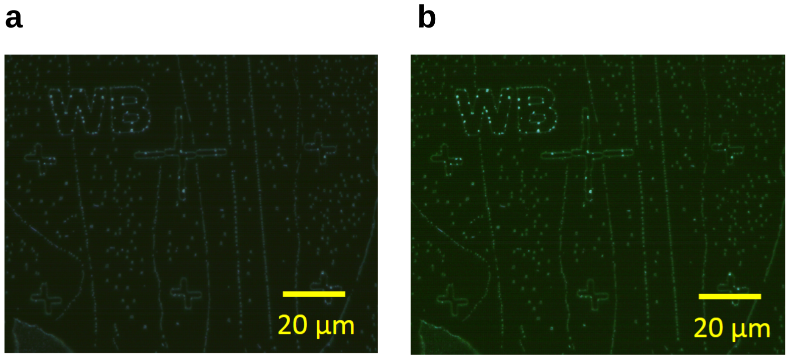

Supplementary Figure 5. **Minimum exposure time for dark field optical images**. (a) 1 msec integration time, (b) 0.5 msec integration time with post capture automatically adjustable brightness and contrast.

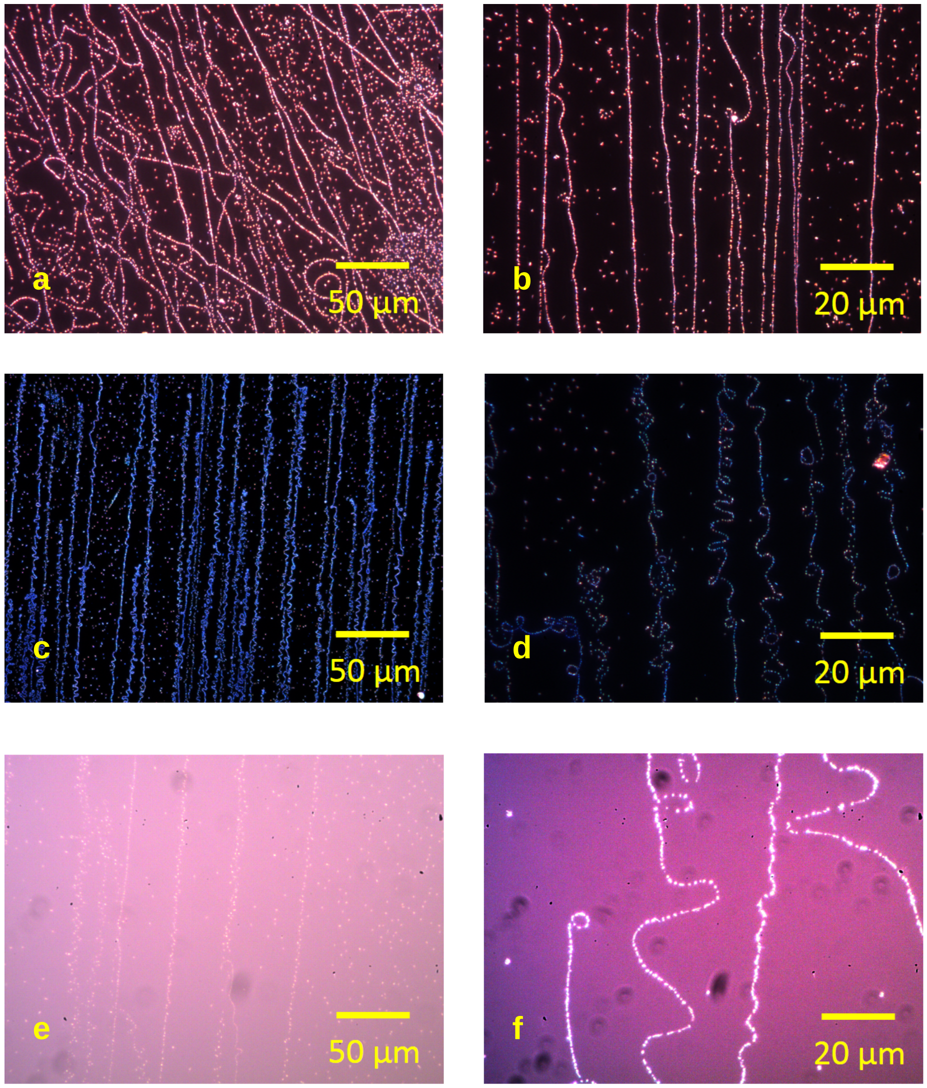

Supplementary Figure 6. **Dark field optical images of marked CNTs with pNBA NCs on different substrates**. (a,b) Al_2O_3, (c,d) Si_3N_4, and (e,f) quartz substrates.

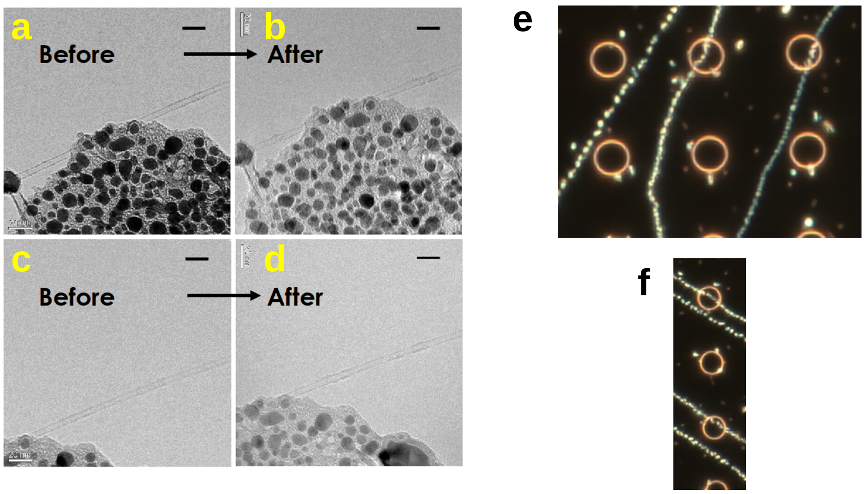

Supplementary Figure 7. Non-invasive imaging. TEM images before (a, c) and long after (b, d) pNBA molecules deposition. Scale bar is 20 nm. (e, f) Dark field optical images of decorated CNTs over TEM grid holes immediately after deposition.

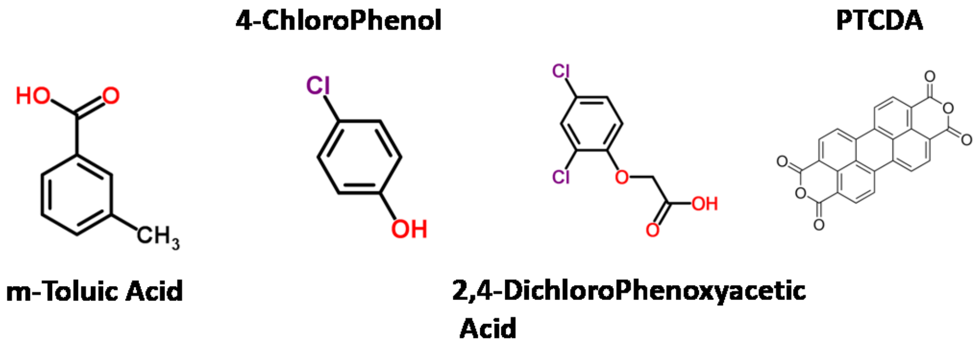

Supplementary Figure 8. **Additional molecules for CNTs marking**. Schematic of the deposited molecules beside pNBA which were adsorbed preferentially to the CNT sidewalls.

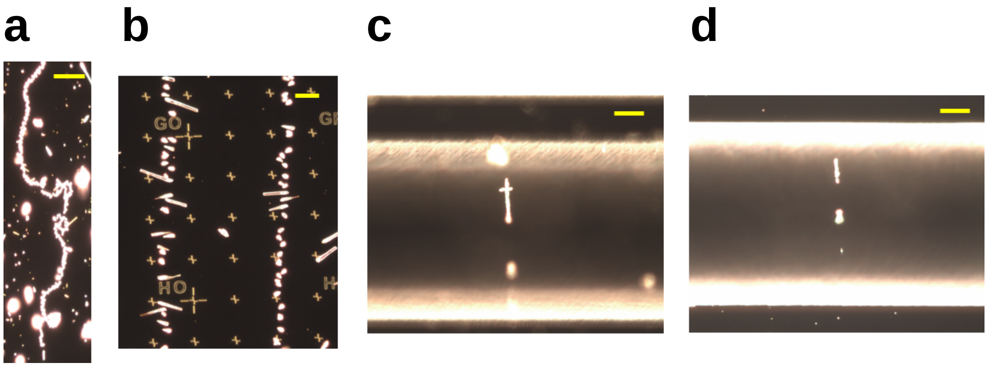

Supplementary Figure 9. **Marking CNTs with m-Toluic Acid NCs**. Dark field images of on-surface (a, b), and suspended (c, d) CNTs. Scale bar - 30 μm.

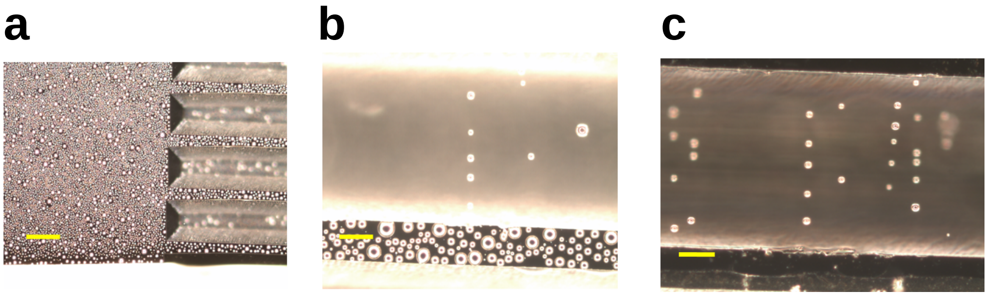

Supplementary Figure 10. **Marking CNTs with 4-ChloroPhenol NCs**. Dark field images of on-surface (a), and suspended (b, c) CNTs. Scale bar - 30 μm.

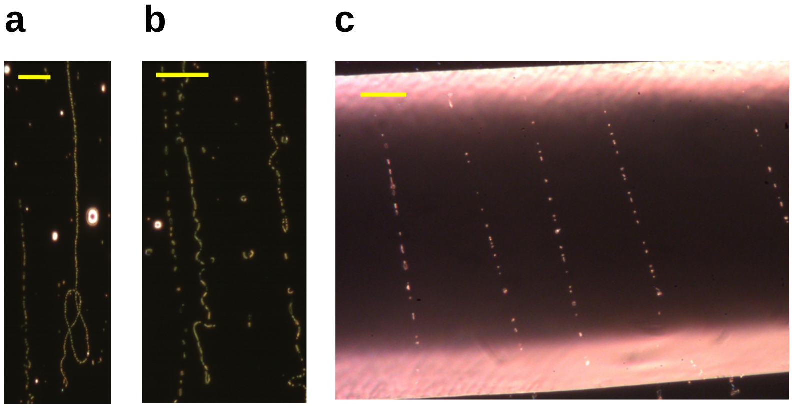

Supplementary Figure 11. **Marking CNTs with 2,4-dichlorophenoxyacetic acid NCs**. Dark field images of on-surface (a, b), and suspended (c) CNTs. Scale bar - 30 μm.

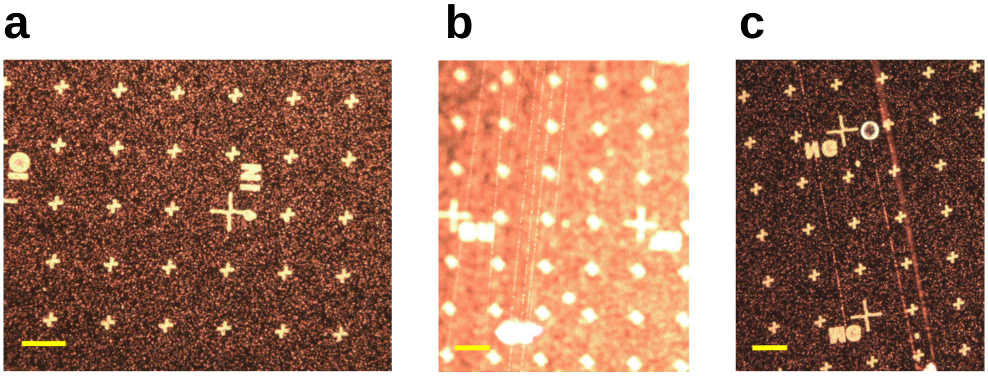

Supplementary Figure 12. **Marking CNTs with PTCDA NCs**. Dark field images of on-surface (a), and suspended (b, c) CNTs (CNTs were grown from elevated catalyst sites). Scale bar - 30 μm.

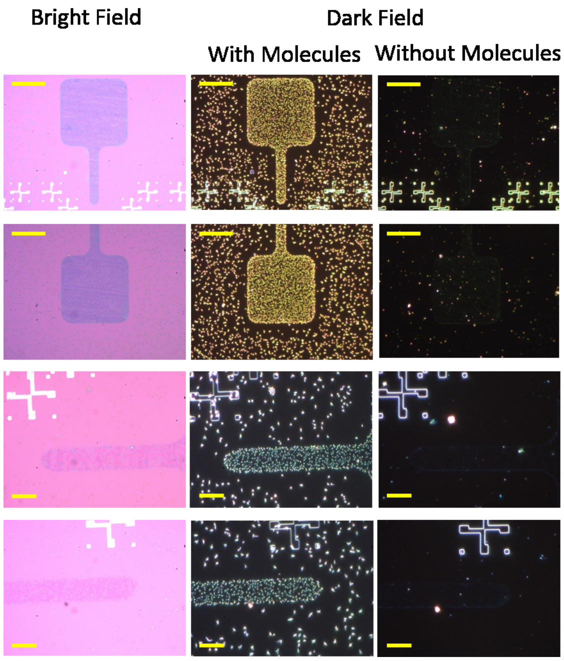

Supplementary Figure 13. **Optical images of single layer CVD graphene on 285 nm** $_2$. (Left column) Bright images, (Middle column) Dark field images with decorated pNBA molecules, (Right column) Dark field images without decorated pNBA molecules. Scale bar - 50 μm (top six images), 15 μm (bottom six images).

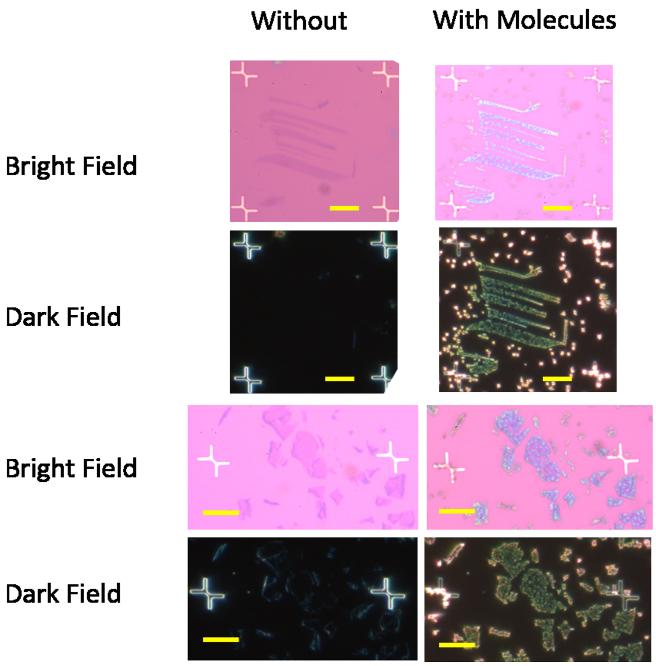

Supplementary Figure 14. **Optical images of single and few layers exfoliated graphene on 285 nm** SiO_2 **with and without pNBA NCs**. Scale bar - 10 μm.

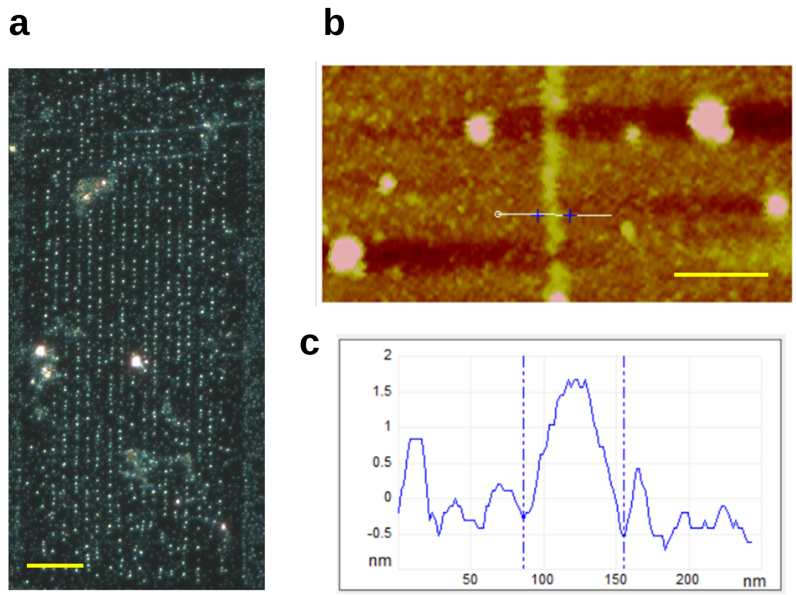

Supplementary Figure 15. **Imaging of graphene nanoribbons**. (a) Dark field optical image of graphene nanoribbons, scale bar - 10 μm, (b, c) AFM image of single nanoribbon and its cross section, scale bar - 200 nm.

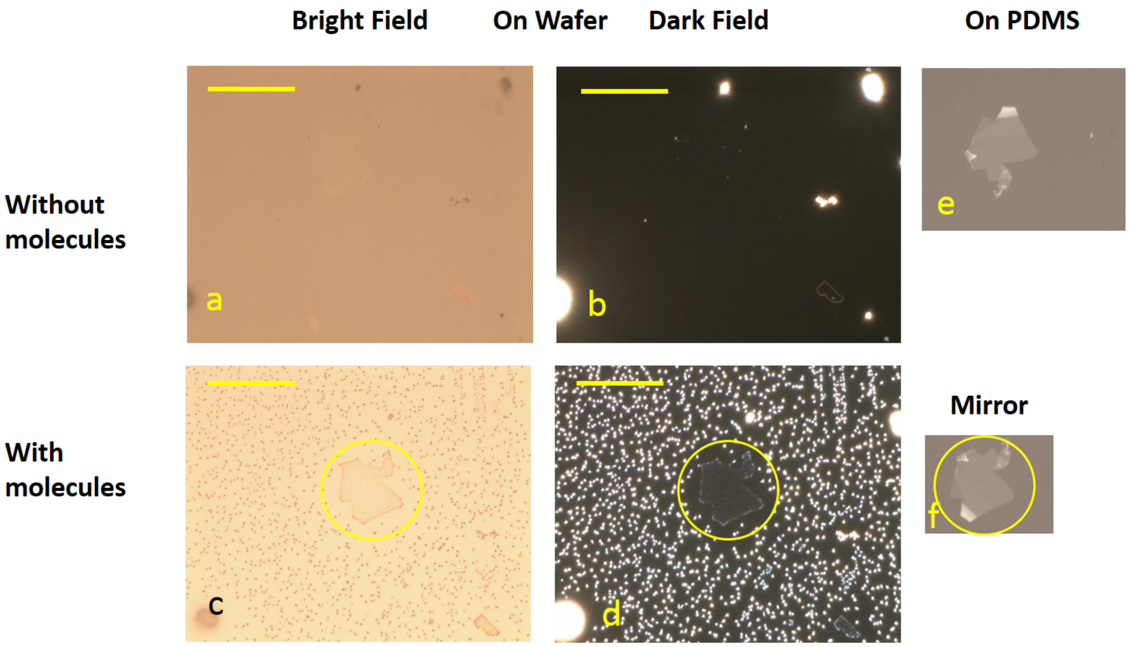

Supplementary Figure 16. **Optical images of single and few layers exfoliated graphene on 10 nm SiO_2 with and without pNBA NCs**. Scale bar - 30 μm.

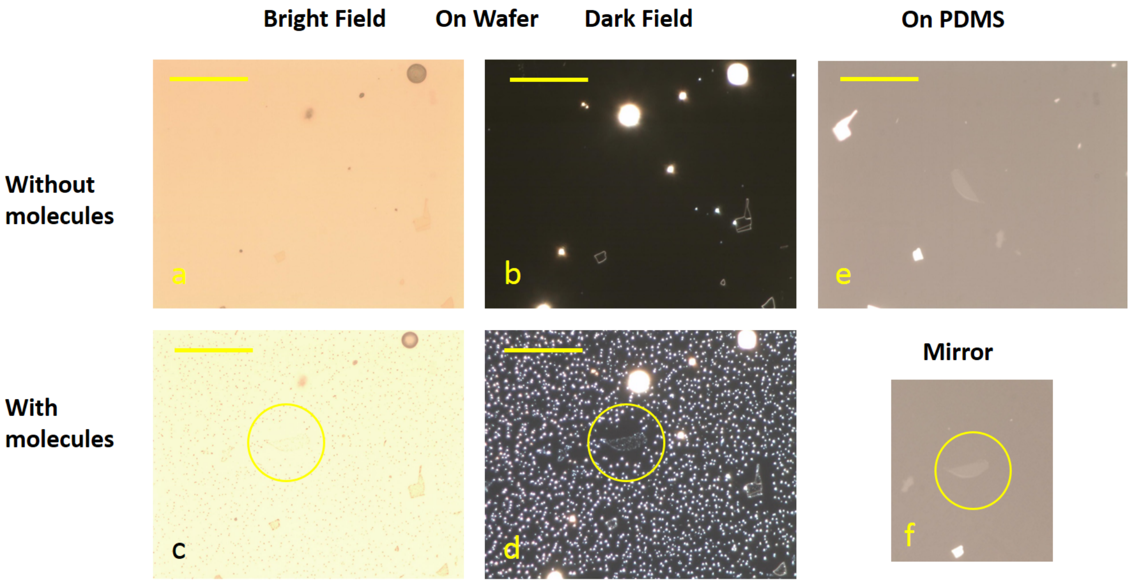

Supplementary Figure 17. **Optical images of single and few layers exfoliated graphene on 10 nm SiO_2 with and without pNBA NCs**. Scale bar - 30 μm.

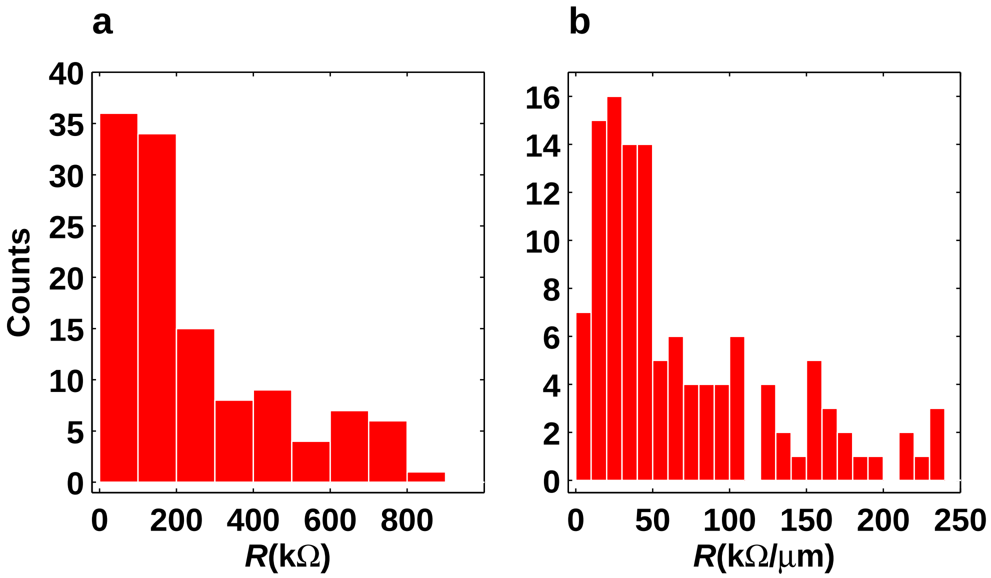

Supplementary Figure 18. **Device quality statistics**. Histograms of the CNTs total resistance (a) and resistance per micron length (b) of the fabricated CNT devices according to the described method.

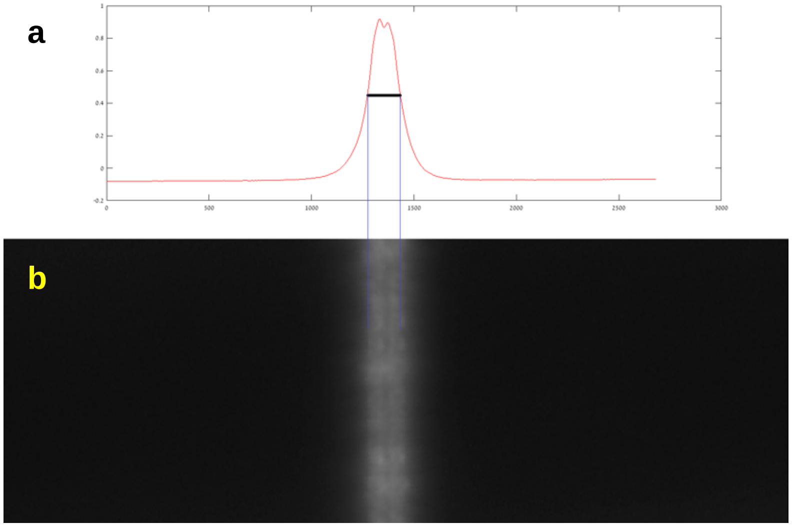

Supplementary Figure 19. **Vibration image procedure**. (a) Normalized optical intensity across vibrating CNT decorated with pNBA NCs. The full width half maximum is marked as black line. (b) Dark field optical image of the central part of the vibrating CNT.

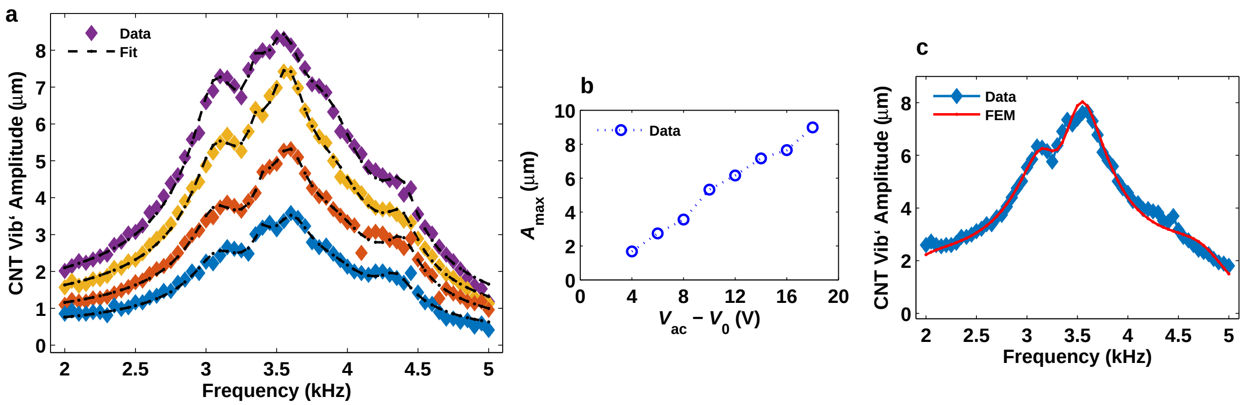

Supplementary Figure 20. **Vibrational analysis**. (a) Amplitude of vibration of CNT versus excitation frequency of a driven piezoelectric actuator. The different colored curves are for different applied voltages V_{ac} of the piezo (8,10,14, and 18 V from bottom to top) . The different black dashed lines are best fit to Lorentzian curves. (b) Maximum amplitude versus the applied ac piezo voltages (subtracted offset bias originated from the electrical setup, V_0). (c) The same data as in (a) for $V_{ac} = 18$ V (blue line) and the resulted fit (red line) according to our finite element model (FEM) discussed in Supplementary Note 7.

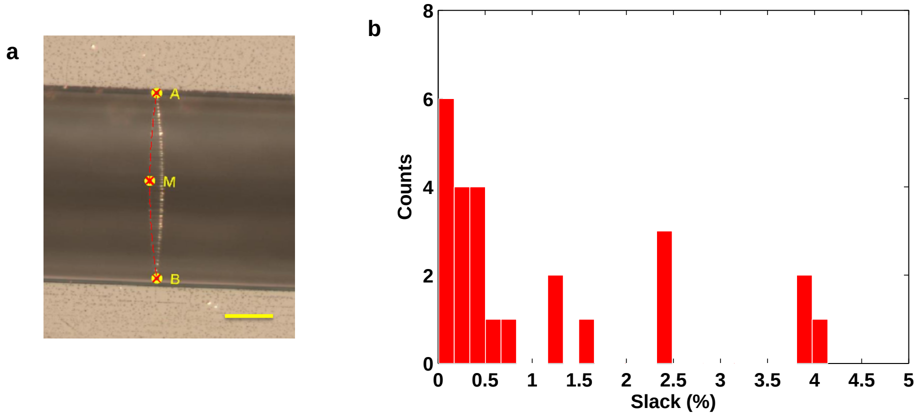

Supplementary Figure 21. **Slack analysis** (a) Dark field image of suspended CNT and the measured length of the tube (red line). Slack, s, is defined as (tube length - AB length)/(AB length). (b) Slack histogram.

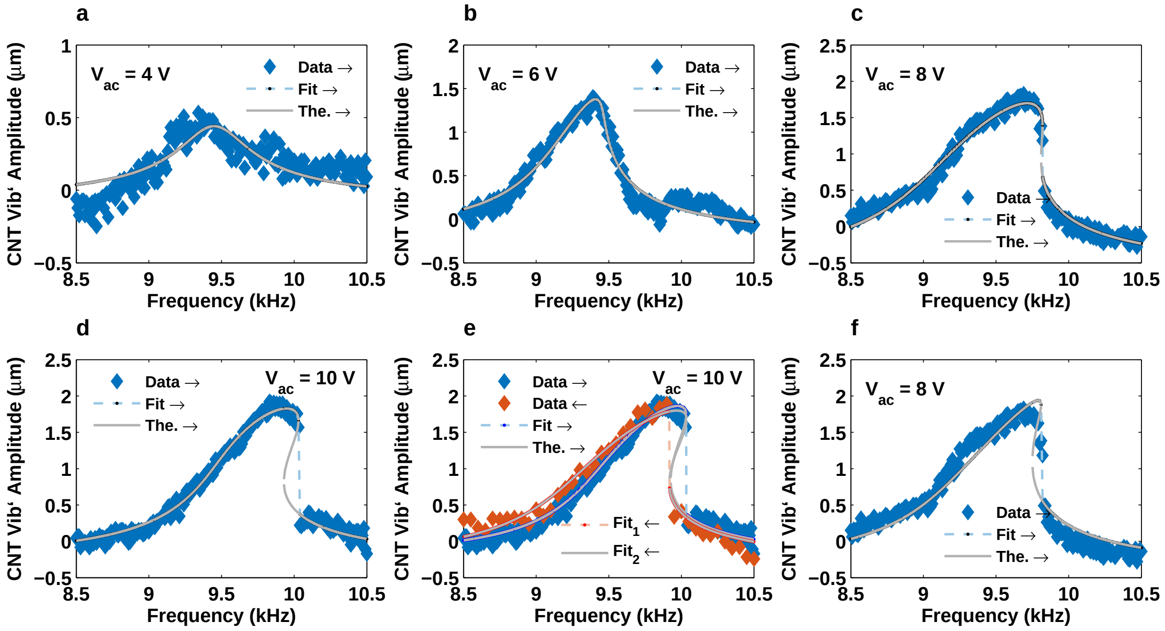

Supplementary Figure 22. **Linear to non-linear behavior**. Amplitude of vibration of CNT versus excitation frequency for different excitation amplitudes, $V_{\rm ac}$'s, as marked inside each panel (a-f). Blue diamonds are data taken for up frequency sweep and red (panel e) for down frequency sweep. The grey lines are the theoretical solutions according to Supplementary Eqs. 33 and 34, and the dashed blue or red lines are the accessible regions for the physical solutions of the theoretic grey lines. (f) The same data as in panel (c), however, the theoretical fit assumes zero nonlinear damping coefficient, i.e., $\gamma_3 = 0$, unlike the rest of the panels (a-e) where $\gamma_3 \neq 0$.

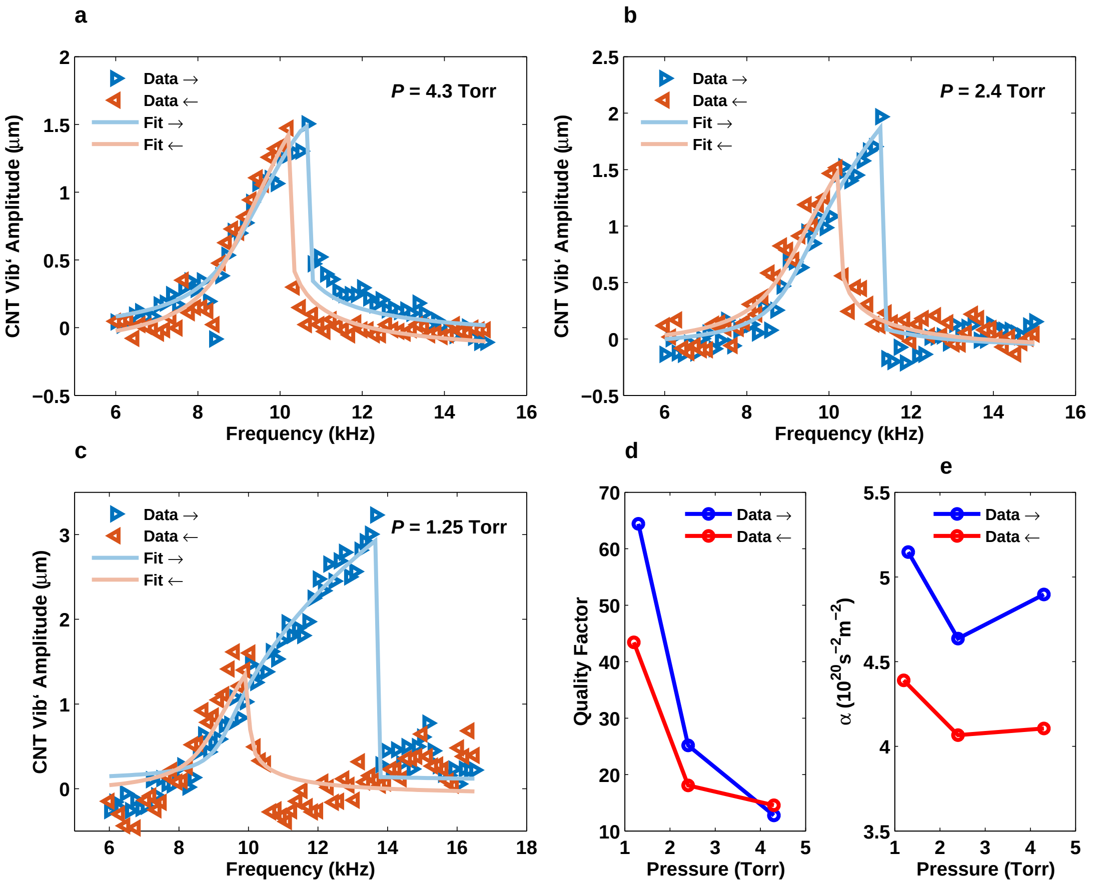

Supplementary Figure 23. **Non-linear and hysteretic behavior**. Amplitude of vibration of CNT versus excitation frequency for different pressures, as marked inside each panel (a-c). Blue triangles are data for up sweep and red triangles for down sweep. The blue and red lines are the theoretical solutions according to Supplementary Eqs. 33 and S34. Extracted quality factor (d) and nonlinear spring constant, α, (e) as function of pressure. The blue points are for up sweep and the red for down sweep.

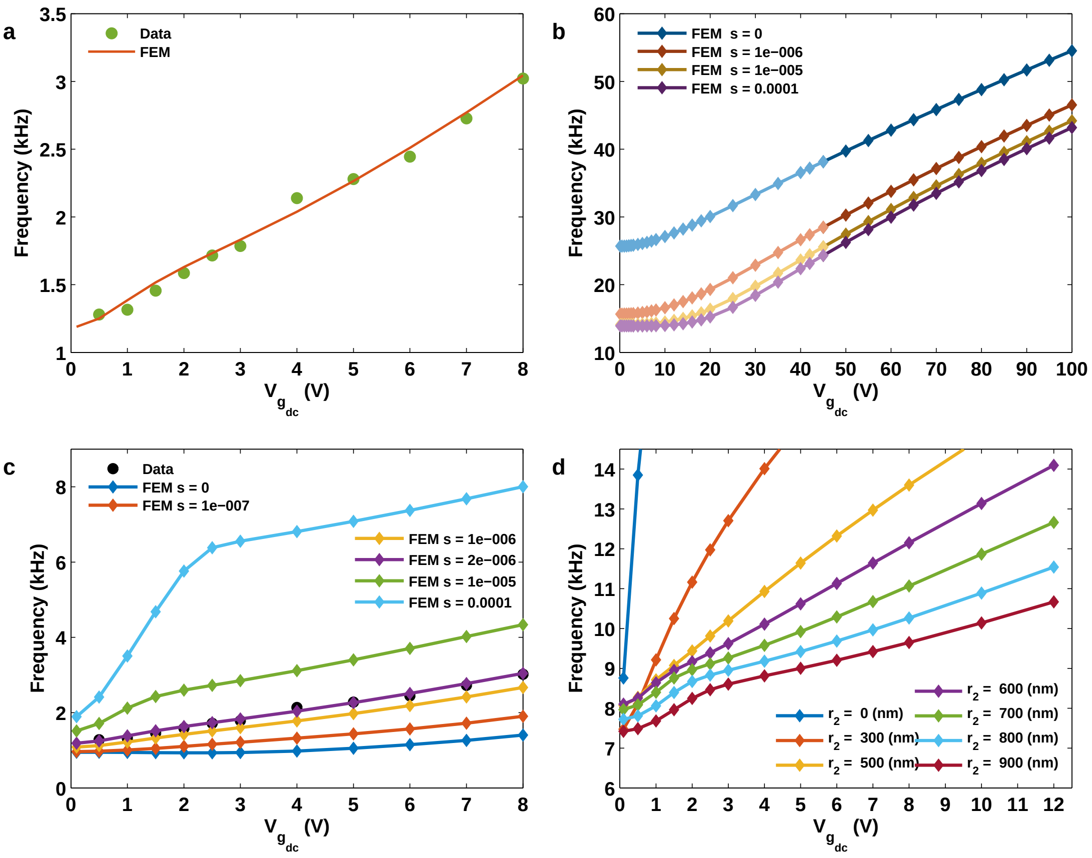

Supplementary Figure 24. Vibrational model for non-homogenous NCs coverage. (a) Resonance frequencies of the first mode under applied dc gate voltages, $V_{g_{dc}}$. Green dots are experimental data and red line is best fit according to our FEM. (b) Theoretical calculations based on our FEM of the first mode versus dc gate voltage for different slack (s) values and homogenous pNBA NCs coverage. (c) Theoretical calculations (FEM) of the first mode versus dc gate voltage for different slack (s) but with nonhomogeneous pNBA NCs coverage. (d) Theoretical calculations (FEM) of the first mode versus dc gate voltage for different maximal amplitude of the random distribution, r_2, and specific values of constant shell, $r_1 = 100\,\text{nm}$ and slack, $s = 0.001$.

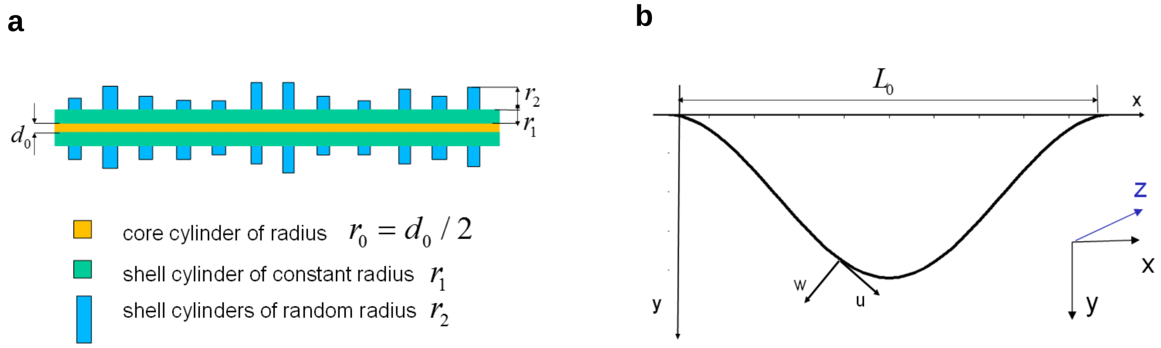

Supplementary Figure 25. CNT model for non-homogenous NCs coverage. (a) Schematic picture of bare CNT with diameter d_0, constant shell of pNBA molecules with radius r_1, and random shell with radius r_2. (b) Slack CNT of length L suspended between trench of length L_0. u and w are the tangential and normal components of the in plane beam displacement, and z presents the out of plane displacement.

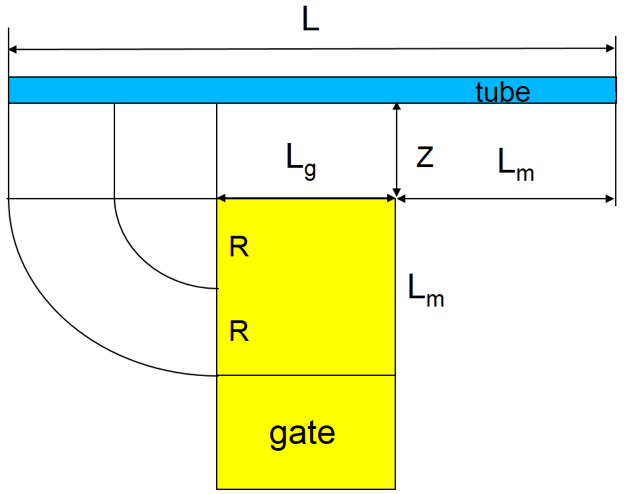

Supplementary Figure 26. CNT-probe capacitive model. Schematic model of the electrostatic coupling between the external probe and the CNT.

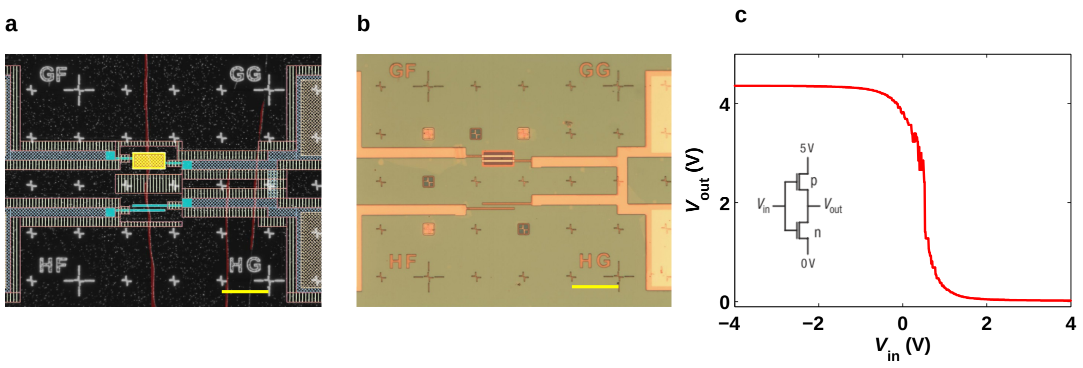

Supplementary Figure 27. Automatic image processing and CNTs based device fabrication. (a) Design layout of CNTs based inverter. The top CNTFET is p type, and the bottom is n type. The yellow rectangular is the passivation layer of SiO_2. (b) The fabricated inverter according to the automatic design layout. Scale bar - 50 μm. (c) Transfer characteristic of an automatically designed inverter based on p and n type CNTFETs. Inset: schematic diagram for the inverter.

Supplementary Note 1 Device fabrication and molecules deposition

Carbon nanotubes (CNTs) were grown using chemical vapor deposition (CVD) at 900°C with 0.5/0.5 SLM flow of H_2/CH_2. The catalyst particles were deposited from ferritin solution onto predefined catalyst pads. Electrical contacts were deposited either before or after CNTs growth. For p-type devices Cr/Au 5/120 nm or Cr/Pt 5/40 nm were deposited[1–4]. For n-type devices four different processes were used. The first was based on 50 nm of

Al deposition capped by 50 nm of Au[5]. The second approach included Ca/Al 30/120 nm deposition[6], and the third consisted of Sc 50 nm metal deposition[7]. The last approach, which was found to be the best, included Cr/Pt 5/40 nm electrical contacts and atomic layer deposition (ALD) of HfO_2 on top of the CNT[8]. The deposition temperature and pressure were 270°C and 620 mTorr, and the HfO_2 thickness was $\approx$ 30 nm.

Powder CNTs were purchased from SWeNT and CoMoCAT^{TM} and were dissolved in chloroform. After tip sonication for 20 min in pulse mode, they were dispersed on silicon wafer. p-nitrobenzoic acid (pNBA) powder was purchased from Fluka. Two main methods were used for deposition of pNBA molecules on the CNTs.

- Deposition on a hotplate: A small metallic tray approximately 1 cm^2 in size is filled with the p-NBA powder and placed near the edge of a 10 cm petri dish. Only this edge is sited on a hotplate, and a glass lid covers the whole petri dish. The hotplate is heated up to 150°C for 15-30 min until p-NBA molecules start to cover the lid. Then, a silicon chip with the CNTs is placed on the cold side of the petri dish, and the molecules condense on the chip. Typically, the condensation period lasts for 90 seconds, but shorter or longer times result with diluted or denser pNBA coverage. A real time video that captured the deposition process along the CNTs is presented in Supplementary Movie 1. The video lasts 30 seconds and it was accelerated by a factor of 20.

- Deposition using furnace: A small furnace made of quartz tube (20 cm length, and 16 mm inner diameter) with a 3 cm heating zone, and Ar gas flow, is preheated to the desired temperature. Then, a small metallic tray with the molecules is placed inside the furnace at the hot zone. After 2 min, the sample with the CNT is placed at the

cold part of the furnace. Typical deposition process was performed with Ar flow of 5 SCCM at 140°C for 4 min.

XRD analysis: X-ray diffraction analysis of p-nitrobenzoic acid powder was carried out by Rikagu MiniFlex X-Ray diffractometer (Cu-Ka1 radiation, $\lambda = 0.540562$ nm). Supplementary Fig. 1 presents the diffraction results. According to these results, it was found that the unit cell of p-nitrobenzoic acid powder is monoclinic with cell parameters of $a = 2.453$ nm, $b = 0.505$ nm, $c = 1.291$ nm, and $\beta = 93.150002°$ (ICDD entry # 00-011-0718).

Supplementary Note 2 Comparison of optical and SEM images

Supplementary Figs. 2a-d present a comparison between dark field optical microscopy images (a, c) and SEM images (b, d) of the same area. It is evident that all the tubes that are seen in the SEM images are also visible by the pNBA marking procedure. Moreover, it happens that CNTs which are seen faintly in SEM images or even not observed, are strongly optically visible (see Supplementary Figs. 2c, d). In all our experiments it was verified that all the tubes that appeared in the SEM images were also seen in the optical images. However, we found that thick CNTs were decorated faster than thin CNTs. This observation hints for more subtle preferences between different tubes, such as tube chirality, or type (semiconducting vs metallic) and the possibility for tube sorting by these pNBA NCs. This issue deserves further research and will be addressed in the near future.

SEM images of suspended CNTs decorated with pNBA NCs are challenging since in high vacuum the pNBA molecules desorb more quickly than at ambient conditions. However, few attempts were successful and Supplementary Fig. 2e depicts a typical example. The tube is suspended, usually vibrates while imaging, and the pNBA molecules form discontinuous NCs chain along its surface. In the attached Supplementary Movie 2, one can observe vibrating of the CNTs under gentle air blowing.

The minimum height of the NC that we can optically image is found by repeated AFM scanning followed by optical imaging. Supplementary Figs. 3a-d present such analysis. On the top left we show dark field optical image of decorated CNTs (Supplementary Fig. 3a) and on the right an enlarged image of the red marked area (Supplementary Fig. 3b). Prior to the optical image we took an AFM scan of the NC marked in yellow circle in Supplementary Fig. 3b (Supplementary Fig. 3c), and present the cross section along the blue line of Supplementary Fig. 3c (Supplementary Fig. 3d). This result, which was found for other cases as well, shows that we can optically image a NC with lateral dimension of less than 100 nm and height of less than 25 nm along the CNT.

The lateral resolution between two adjacent CNTs was found to be ≈ 250 nm. Supplementary Fig. 4a presents an optical image of few marked CNTs with pNBA NCs. If we enlarge the area marked by a red square we notice two parallel CNTs which are very close to each other, as seen in Supplementary Fig. 4c. Supplementary Fig. 4b shows SEM image of the whole region, where, Supplementary Fig. 4d presents an enlarge SEM image of the red dashed square area of Supplementary Fig. 4c. The three circles in both images (Supplementary Figs. 4c and 4d) assist in finding the exact mapping between the two images. The distances between the tubes can be accurately measured from the SEM image (Supplementary Fig. 4d). Hence, it is clearly seen from the optical image (Supplementary Fig. 4c) that for tube separation which is larger than 260 nm the two CNTs are distinct. Supplementary Figs. 5a, b show the minimum integration time required for optical imaging of the pNBA NCs. As expected, this time is much shorter in comparison to previous optical methods and found to be less than 1 msec for an area of $120 \times 120\,\mu\text{m}^2$. This imaging technique is applicable to a variety of substrates including high-k dielectrics, insulators, and transparent substrates. Supplementary Figs. 6a-f show few examples for such optical imaging.

TEM analysis: Commercial TEM grids with thin silicon nitride mesh film 200 nm thick, and 2 μm diameter holes were used. Catalyst solution was deposited on the circumference of the TEM grid and CNTs were grown using CVD tool. Imaging was performed using 120 kV, LaB6 emitter equipped, FEI T12 G2 TEM, before and sufficiently long time after (two weeks) pNBA deposition. The results are depicted in Supplementary Figs. 7a-d. It is evident that images of the same area before and long after deposition are essentially unchanged, and there is no evidence of molecule residue. Optical images just after deposition verify that all the tubes which bridge the grid holes are indeed decorated with pNBA NCs (Supplementary Figs. 7e, f).

Supplementary Note 3 Marking with other molecules

Beside pNBA molecules, additional candidates were tested. Those which showed partial or full success are discussed in this section. Several features were found to be essential for the marking procedure. The first, and probably the most important is the existence of at least one benzene ring in the deposited molecules. The second, is its capability to diffuse on the cold substrate at ambient conditions, and the last, is the fact that they crystalize to solid phase at room temperature. Below we present our results in more detail. Supplementary Fig. 8 presents the schematic structures of four additional molecules that have shown partial or full success of decorating the CNTs beside the pNBA molecule.

The first molecule, m-Toluic acid, has a similar structure of benzene ring and carboxylic acid group as pNBA. However, the deposition process is different. For m-Toluic acid, the molecules were evaporated from the liquid phase and the resulted NCs which decorated on-surface CNTs have a needle like shape (Supplementary Figs. 9a, b). They succeeded to crystalize along suspended CNTs as well (Supplementary Figs. 9c, d) however in nonhomogeneous fashion.

The second molecule is 4-Chlorophenol which melts at relatively low temperature (40°C) and evaporates very quickly. When we deposited these molecules, again from the liquid phase, onto SiO_2 substrates with CNTs, they formed spherical shapes all over the surface without any preferential adsorption toward the CNTs (Supplementary Figs. 10a, b). Nevertheless, when deposited over suspended CNTs, these molecules formed spherical NCs along the CNTs in a pearl like chain (Supplementary Figs. 10b, c). After few minutes, these NCs disappeared both from the substrates and the suspended CNTs.

The third molecule, 2,4-dichlorophenoxyacetic acid, again has benzene ring and carboxylic acid group in its structure. These molecules are deposited from the liquid phase and adsorbed preferentially to both on-surface and suspended CNTs, as depicted in Supplementary Figs. 11a-c. Nevertheless, the decorated NCs are less continuous along the suspended CNTs with comparison to pNBA NCs, and they did not mark all the on-surface CNTs. This result is very interesting, since it may suggest some sorting mechanism for these molecules, but further research is required to unveil it quantitatively.

The last molecule is 3,4,9,10- perylene tetracarboxylic dianhydride (PTCDA) which has several benzene rings. These molecules were deposited directly from the solid phase, as pNBA molecules. However, since they are very big (high molecular mass with respect to pNBA), when they were adsorbed onto the substrate, they could not diffuse much and formed NCs all over the substrate, without any preference (Supplementary Fig. 12a). However, when deposited over suspended CNTs, PTCDA molecules formed NCs along the CNTs and made them optically visible (Supplementary Figs. 12b, c).

Supplementary Note 4 Marking single and few layers graphene

This marking procedure is applicable also for graphene samples, as may be expected. The method is effective for exfoliated graphene, CVD graphene, nanoribbons, and also for making

graphene optically visible on different substrates which do not support constructive interference as exists for example for graphene on 285 nm SiO_2[9].

Supplementary Fig. 13 clearly shows CVD graphene patterned in the shape of electrode on 285 nm SiO_2. In the bright field images single layer graphene is visible thanks to the oxide thickness. In the dark field images the graphene patterns are visible only due to the decoration of the NCs. Supplementary Fig. 14 depicts similar results for exfoliated single and few layers graphene. Again, the visibility of the graphene flakes is clearly demonstrated for the decorated samples.

Graphene nanoribbons play an important role in graphene transistors. The transversal constriction imposes formation of energy band gap along the graphene flake making it applicable for transistor operation. Usually, since these nanoribbons are very narrow, it is hard to find and image them even on 285 nm SiO_2. Supplementary Fig. 15a presents dark field image of several nanoribbons that we have fabricated using ebeam lithography and decorated with pNBA NCs. The ribbons widths are approximately ≈ 50 nm and few nm in height (Supplementary Figs. 15b, c). As evident, the NCs form a chain like structure along the graphene nanoribbons.

In many studies single or few layer graphene are deposited on thin or high-k dielectrics. In such cases the graphene flakes are not optically visible and device fabrication imposes great challenges. Harnessing our staining technique to these cases is of great importance. Supplementary Figs. 16 and 17 present successful attempts towards this goal. In these figures single or few layers graphene were exfoliated on PDMS mask and deposited on substrates with 10 nm SiO_2. In the optical bright or dark field images the graphene flakes are not visible if the pNBA NCs are absent. However, when we deposited the molecules on the substrates, the flakes became optically visible, both by bright and dark fields microscopy. This possi-

bility significantly simplifies the fabrication and characterization of graphene based devices where multiple layers of graphene and hexagonal boron nitride are involved.

Supplementary Note 5 Sublimation rate

In this section we derive the temporal dependence of the sublimation process of pNBA molecules which desorb from the CNT surface. This analysis follows the study of Sambles *et al.*[10]. The sublimation rate depends on the partial pressure of the pNBA molecules in the gas phase in the vicinity of the nano-crystal (NC). Assuming spherical geometry of the NC, with radius r, one can employ Kelvin equation (Supplementary Eq. 1) for evaluating the partial pressure, P_r, in the gas phase adjacent to the NC surface. In this relation

$$P_r = P_\infty \exp\left(\frac{2\gamma M_r}{RT\rho r}\right) \tag{1}$$

P_∞ is the partial pressure above flat surface, γ is the surface energy of the NC, ρ is its mass density, M_r is its molecular weight, T the temperature, and R is the gas constant. Defining n_v as the number of molecules leaving a unit area of the NC per second, and V_a as the volume of a single molecule, results with relation between the temporal change of the NC radius and the outgoing particle flux: $dr/dt = n_v V_a$. Kinetic theorem tells us that the number of vapour molecules colliding the NC surface per unit area per second is $n_c = n\bar{c}/4$ where n is the number of molecules per unit volume in the vapour, and $\bar{c} = \sqrt{8RT/\pi M_r}$ is their average speed.

It is assumed that the rates of condensation and evaporation are independent, thus, at dynamic equilibrium, $n_v = \alpha n_c$, where α is the fraction of colliding particles which become integral part of the solid phase. Combining these relations, and assuming that the vapour

behaves as a mono-atomic perfect gas, i.e., $n = P/k_BT$, yields

$$\left.\frac{dr}{dt}\right|_r = \frac{1}{4}\alpha n V_a \sqrt{\frac{8RT}{\pi M_r}} = \sqrt{\frac{M_r}{2\pi R \rho^2 T}}\alpha P_r. \tag{2}$$

When the radius of curvature is $r \to \infty$ the last equation reduces to

$$\left.\frac{dr}{dt}\right|_\infty = \frac{1}{4}\alpha n V_a \sqrt{\frac{8RT}{\pi M_r}} = \sqrt{\frac{M_r}{2\pi R \rho^2 T}}\alpha P_\infty. \tag{3}$$

Dividing the last two equations, results with a differential equation for $r(t)$ which describes the sublimation process of the pNBA NCs:

$$\left.\frac{dr}{dt}\right|_r = \left.\frac{dr}{dt}\right|_\infty \frac{P_r}{P_\infty} = \left.\frac{dr}{dt}\right|_\infty \exp\left(\frac{M_r}{R\rho}\frac{1}{T}\frac{2\gamma}{r}\right). \tag{4}$$

where the ratio between the two partial pressures was replaced by the Kelvin equation (Supplementary Eq. 1).

The surface energy of a NC is lower than its bulk value, and depends on the NC size[11]. This arises from the reduction of the cohesive energy due to the increase in surface atoms compared with bulk atoms as the size decreases. A common expression which takes this effect into account reads the following:

$$\gamma(r) = \gamma_0\left(1 - \frac{2h}{r}\right), \tag{5}$$

where γ_0 is the bulk value, and h is the size of the unit cell of the sublimed material. Since

r decreases with time, Supplementary Eqs. 4 and 5 reduce to

$$\left.\frac{dr}{dt}\right|_r = -A\exp\left[B\left(1-\frac{2h}{r}\right)\frac{1}{r}\right], \tag{6}$$

which has the following solution

$$\int_{r_0}^{r}\frac{dr}{\exp(B(1-\frac{2h}{r})\frac{1}{r})} = -At \tag{7}$$

where r_0 is the initial radius of the NC, $A = dr/dt|_\infty$, and $B = 2M_r\gamma_0/\rho RT$. Supplementary Eq. 7 is solved numerically and fitted to the experimental sublimation data, as depicted, for example, in Figs. 2c, d. Although this analysis is only an approximation for the real problem, the resulted values for h and γ_0 are in good agreement with their previously reported values. h and γ_0 should be a weighted average of the NC facets, and theoretically should be $0.5 < h < 1$ nm and $30 < \gamma_0 < 60\,\mathrm{Nm}^{-1}$. For example, the fitted data of Fig. 2c (red line) corresponds to $h = 0.55 \pm 0.05$ nm, and $\gamma_0 = 25 \pm 5\,\mathrm{Nm}^{-1}$, which are in good agreement with the theoretical results.

Supplementary Note 6 Electrical measurements

One of the most rewarding observation of this study is the simplicity of making CNTs devices utilizing this method. The marking procedure can be performed either for a complete circuit, in order to identify the locations of the existing tubes, or, for the design of a new circuit according to the optical images. We have used this method and fabricated more than 100 CNT based devices, with more than 95% success rate. The high yield is attributed to the knowledge of the exact location of the complete CNT network and the possibility to place our electrodes on optimal location, where the tubes are straight, far from other tubes,

and do not split to additional tubes. Supplementary Figs. 18a, b depict two histograms of the total resistance and per micron length of the CNT devices that were fabricated and measured during this study assisted by the described method. The left histogram includes CNT devices with variety of metallic electrodes, and lengths (most of them are above 5 μm in length). As evident from the graph, more than 50% of the devices have total resistance which is less than 200 kΩ, and if we normalize the resistance per micron length we obtain Supplementary Fig. 18b. Here, we notice that more than 50% of the devices have resistance per micron which is less than 50 kΩ. These results are extremely good and are well within the high-end group of existing devices.

Supplementary Note 7 Vibrational analysis

The vibrational modes of doubly clamped suspended CNT without slack are found from the solutions of Euler-Bernoulli beam equation[12]. However, most of our CNTs have slack, as can be extracted directly from the optical images. Supplementary Figs. 21a, b show how we measure the slack of a typical suspended CNT and the resulted slack distribution, respectively. With slack, the solution for the Euler-Bernoulli beam equation is more complex, and usually requires numerical analysis[13]. Since our tubes are also decorated with pNBA NCs, which form non homogenous shell around the original CNT, finite element algorithm is mandatory. The novel procedure we have developed is outlined below:

A CNT with a core cylinder of radius $r_0 = d_0/2$ (see Supplementary Fig. 25a) is surrounded by pNBA NCs which can be modeled by two parts of a cylindrical shell: a) a cylinder of constant radius r_1, and b) cylindrical segments of random heights, r_2. Under slack conditions, the CNTs can have either buckling form, parabolic form, or others. Let us assume for the moment that the CNT is a curved planar beam which has a buckling form of the following shape: $y(x) = b(1 - \cos 2\pi x/L_0)$, where the different symbols are drawn schematically in

Supplementary Fig. 25b.

The differential equations, describing the in-plane vibration of a curved planar beam are given by[14]

$$\rho_v S\frac{\partial^2 u}{\partial t^2} + \rho_v S\gamma\frac{\partial u}{\partial t} - \frac{\partial}{\partial p}\left(ES\left(\frac{\partial u}{\partial p} - kw\right)\right) = \mathfrak{F}_1 \exp(i\omega t)$$
$$\rho_v S\frac{\partial^2 w}{\partial t^2} + \rho_v S\gamma\frac{\partial w}{\partial t} + \frac{\partial^2}{\partial p^2}\left(EI\frac{\partial^2 w}{\partial p^2}\right) - ES\left(\frac{\partial u}{\partial p} - kw\right)k - \frac{\partial}{\partial p}\left(\sigma_0\frac{\partial w}{\partial p}\right) = \mathfrak{F}_2 \exp(i\omega t), \quad (8)$$

where $u = u(p,t)$, and $w = w(p,t)$. Here (u,w) are the tangential and normal components of the beam displacement, p is the coordinate along the beam, and t represents time. k is the initial curvature of the beam, and γ is the damping coefficient. The other parameters, E, ρ_v, S, and I are standard notations of Young modulus, mass density, cross section area and moment of inertia, respectively. $\mathfrak{F}_1$ and $\mathfrak{F}_2$ are the tangential and normal projections of the oscillatory driving force at angular frequency ω. For doubly clamped beam the in plane boundary conditions are: $u(0,t) = u(L,t) = w(0,t) = w(L,t) = \frac{\partial w(0,t)}{\partial p} = \frac{\partial w(L,t)}{\partial p} = 0$, where L is the beam length. The local strain and curvature change satisfy the same relation as for shallow shells[14], i.e.,

$$\epsilon = \frac{\partial u}{\partial p} - kw, \qquad \chi = -\frac{\partial^2 w}{\partial p^2}. \quad (9)$$

For the out of plane vibration (orthogonal to the x-y plane) the following equations hold[15]:

$$EIk\left(\frac{\partial^2 \nu}{\partial p^2} - k\beta\right) + \frac{\partial}{\partial p}\left(GJ\left(\frac{\partial \nu}{\partial p} - k\beta\right)\right) = \rho_v J\frac{\partial^2 \beta}{\partial t^2}$$
$$\rho_v S\frac{\partial^2 \nu}{\partial t^2} + \frac{\partial^2}{\partial p^2}\left(EI\left(\frac{\partial^2 \nu}{\partial p^2} - k\beta\right)\right) - \frac{\partial}{\partial p}\left(GJk\left(\frac{\partial \beta}{\partial p} + k\frac{\partial \nu}{\partial p}\right) + \sigma_0\frac{\partial \nu}{\partial p}\right) = \mathfrak{F}_3 \exp(i\omega t), \quad (10)$$

where $\nu = \nu(p,t)$, and $\beta = \beta(p,t)$. Here (ν, β) are the displacement in the z direction, and the axial rotation[15], respectively. G is the shear modulus, J is the moment of rotation, and

F_3 is the out of plane projection of the oscillatory driving force at angular frequency, ω. The relevant boundary conditions for doubly clamped beam are: $\nu(0,t) = \nu(L,t) = \beta(0,t) = \beta(L,t) = \frac{\partial \nu(0,t)}{\partial p} = \frac{\partial \nu(L,t)}{\partial p} = 0$.

In order to solve these equations, first, we eliminate the time from the two sets of equations (using separation method) and obtain two boundary-value problems. Afterwards, we solve the boundary-value problems using finite element method (FEM) with Galerkin procedure[14]. The cubic (Hermit) FEM approximation was used for $w(p)$ and $\nu(p)$, and linear FEM approximation was used for $u(p)$ and $\beta(p)$[16]. The experimental vibrational modes were automatically measured by matlab code which captured the dark field image of the vibrating tube and analyzed its shape for each frequency at the measured range. Gaussian fit was employed for fitting the intensity profile and the vibrating amplitude was extracted from the full width half max (Supplementary Fig. 19). Fig. 5e and Supplementary Fig. 20c depict two examples of the experimental data and the obtained fits according to the FEM. The slack of each tube was extracted from the optical image, and the two fitting parameters were only r_1 and r_2. The random distribution of the outer shell is essential for obtaining excellent agreement between the finite element approach and the experimental data.

For the electrostatic actuation, both dc and ac electrical forces are applied between the vibrating tubes and the metallic probe, which serves as an external gate. The constant voltage between the tube and the gate creates tension along the tube, which affects its resonance frequencies. Supplementary Fig. 24a presents the frequency increase of the fundamental resonance mode as the external dc gate voltage increases. As before, since the tube contains nonhomogeneous distribution of NCs, a simple phenomenological model which interpolates between the bending behavior at low external fields and the elastic behavior at high biases will not be adequate[17]. However, our FEM which takes into account prestressed beam con-

figuration, is appropriate for these cases, as well. Again, the original slack is found from the optical image, and with only two fitting parameters, r_1 and r_2, we could nicely fit the experimental results, for the whole bias range (Supplementary Fig. 24a). The electric force which is exerted on the tube depends on the capacitance coupling between the gate and the CNT. Since the external probe is shorter than the tube length the standard expression for the capacitance between the tube and the metallic plane, $C_{\mathrm{g}}(z) = 2\pi\epsilon_0 L/\log(2z/r_0)$, where z is the vertical distance between the tube and the gate, is not adequate. A common approach for the current configuration is based on approximating the metallic probe to cut cone, and adding together the contributions of the face and side wall capacitances as plotted in Supplementary Fig.26. Exact electrostatic solution using COMSOL simulation agrees very well with our approximate model. The total capacitance and the total electric force are given by

$$\begin{aligned}
&C_g = C_1 + 2C_2, \\
&C_1 = \frac{2\pi\epsilon_0 L_g}{\log(2z/r_0)}, \\
&C_2 = \frac{2\pi\epsilon_0 L_m}{\log(2z_m/r_0)}, \\
&L_m = (L - L_g)/2, \qquad z_m = z + \pi R/2, \qquad R = L_m/2, \\
&F_{dc} = \frac{1}{2}\frac{dC_g}{dz}V_g^2(dc).
\end{aligned} \tag{11}$$

The residual tensions which are tuned by the applied dc voltage, as well as the vibrational modes are calculated by the FEM. The resulted resonances of the first mode as a function of the gate voltages are plotted in Supplementary Fig. 24a, with r_1 and r_2 as the two fitting parameters. As evident, the agreement between the experimental data and the theoretical prediction is excellent, but the data do not follow the expected behavior of homogenous

CNT[13]. Supplementary Fig. 24b presents the calculated vibrational modes by our FEM for homogenous CNT with constant shell but without random coverage. The different curves are for different slacks, but in all of them one can nicely observe the bending regime for small dc voltages, the catenary regime for intermediate dc voltages, and the sublinear behavior for high gate voltages, which arises from tube stretching[13]. However, completely different behavior is found when slack and non homogenous distribution is introduced. Supplementary Fig. 24c depicts the calculated vibrational modes for CNTs with different slack but the same r_1 and r_2, where, Supplementary Fig. 24d presents the calculated vibrational modes for CNTs with different random distribution, r_2, but the same r_1 and slack. As evident, for increasing slack (s) and amplitude of the random shell (r_2) the calculated vibrational modes differ significantly from the prediction for the homogenous tube[13].

For large ac excitations the frequency response reflects non linear behavior and hysteretic shape. Examples are depicted in Fig. 6 and Supplementary Fig. 22. A common model which describes such phenomena named Duffing oscillator[18], presents a lumped model for the beam resonator. Below we present a derivation of this model for the studied CNTs under high dc gate voltage, i.e., in the limit where stretching is stronger than bending, and restrict ourselves to in-plane motion. First, we need to solve the linear Euler-Bernoulli beam equation,

$$EI\frac{\partial^4 u(x)}{\partial x^4} - T_0\frac{\partial^2 u(x)}{\partial x^2} = \rho_v A\omega^2 u(x), \tag{12}$$

where u is the in-plane motion, and T_0 is the prestress tension, or residual tension arising from the dc gate voltage. For convenience, we choose the following boundary conditions: $u(-L/2) = u(L/2) = u'(-L/2) = u'(L/2) = 0$, and divide Supplementary Eq. 12 by T_0 to

yield

$$\xi^2 \frac{\partial^4 u(x)}{\partial x^4} - \frac{\partial^2 u(x)}{\partial x^2} = \lambda^2 u(x), \tag{13}$$

where $\xi^2 = EI/T_0$, and $\lambda^2 = \rho_v A\omega^2/T_0$. The solution for Supplementary Eq. 13 is given by[12]

$$u(x,t) = u_t(t) \left(\cos(k_1 x) - \frac{\cos(k_1 L/2)}{\cosh(k_2 L/2)} \cosh(k_2 x) \right), \tag{14}$$

where,

$$k_1 = \frac{1}{\sqrt{2}\xi} (\sqrt{1 + 4\xi^2\lambda^2} - 1)^{1/2} \tag{15}$$

$$k_2 = \frac{1}{\sqrt{2}\xi} (\sqrt{1 + 4\xi^2\lambda^2} + 1)^{1/2}. \tag{16}$$

Using the following relation, $\xi^2 k_2^2 = 1 + \xi^2 k_1^2$, and the secular equation

$$k_1 \cosh(k_2 L/2) \sin(k_1 L/2) + k_2 \cos(k_1 L/2) \sinh(k_2 L/2) = 0 \tag{17}$$

we receive an implicit equation for k_1. For high dc gate voltages, $\xi^2 = EI/T_0 \ll 1$, thus we can expand Supplementary Eq. 17 in power of ξ, and find the leading terms of k_1, k_2, $\lambda = k_1 k_2 \xi$, and finally the resonance frequency, $f_1 = \omega_1/2\pi$, of the fundamental mode, i.e.,

$$k_1 = \frac{\pi}{L} \left(1 + 2\xi + 4\xi^2\right) + O(\xi^3) \tag{18}$$

$$k_2 = \frac{1}{\xi} \left(1 + \frac{\pi^2\xi^2}{2L^2} + \frac{2\pi^2\xi^3}{L^3} + O(\xi^4) \right) \tag{19}$$

$$f_1 = \frac{\lambda}{2\pi} \sqrt{\frac{T_0}{\rho_v S}} = \frac{1}{2L} \sqrt{\frac{T_0}{\rho_v S}} \left(1 + \frac{2\xi}{L} + \frac{8 + \pi^2}{2} \frac{\xi^2}{L^2} + O(\xi^3) \right). \tag{20}$$

Next we derive the lump Hamiltonian of the vibrating beam. The kinetic energy is given by

$$E_k = \frac{1}{2}\rho_v S \int_{-L/2}^{L/2} \left(\frac{\partial u(x)}{\partial t}\right)^2 dx = \frac{1}{4}(\dot{u}_t)^2 L\rho_v S(1-2\xi) + O(\xi^3), \tag{21}$$

and the potential energy is the following

$$\begin{aligned} E_p =& \tfrac{1}{2}\int_{-L/2}^{L/2}\left(EI\left(\tfrac{\partial^2 u(x)}{\partial^2 x}\right)^2 + \left(T_0 + \tfrac{ES}{2L}\int_{-L/2}^{L/2}\left(\tfrac{\partial u(x)}{\partial x}\right)^2\right)\left(\tfrac{\partial u(x)}{\partial x}\right)^2\right)dx \\ = & \quad \tfrac{u_t^2\pi^2 EI}{2\pi^2 L^3\xi} + \tfrac{2u_t^2 EI\pi^2}{L^3} + \tfrac{u_t^2 EI\pi^4}{4L^3} + \tfrac{u_t^2\pi^2 T_0}{4L} + \tfrac{u_t^4 ES\pi^4}{16L^3} + O(\xi), \end{aligned} \tag{22}$$

where T_0 is the residual tension. The equation of motion within the lump model is written as follows:

$$\frac{1}{2}\ddot{u}_t L\rho S = -\frac{\partial E_p}{\partial u_t} + F_{dc} + F_{ac}, \tag{23}$$

where F_{dc} and F_{ac} are the external dc and ac applied electric forces. When the beam displacements are not negligible with respect to the distance between the tube and the external probe (z), one should expand the external dc force in powers of u. Such expansion together with rearrangement of Supplementary Eq. 23 yield the following expression:

$$\ddot{u}_t + \omega_0^2 u_t + \frac{E\pi^4}{2\rho_v L^4}u_t^3 = F_0 + F_1 u_t + F_2 u_t^2 + F_3 u_t^3 + F_{ac}/m, \tag{24}$$

where $m = L\rho S/2$ and

$$\omega_0^2 = \left(\frac{T_0}{\rho_v S}\right)\left(\frac{\pi^2}{L^2} + \frac{2\pi^2}{L^2}\sqrt{\frac{EI}{T_0 L^2}} + \frac{8\pi^2}{L^2}\frac{EI}{T_0 L^2} + \frac{\pi^4}{L^2}\frac{EI}{T_0 L^2}\right). \tag{25}$$

In the calculations below, we have used the full expression for the different $F_i's$ in Supplementary Eq. 30 up to the fourth powers in ξ. However, for the simplicity of typing we note

only the leading terms of these expressions for the approximated case where $L_g \to L$ and $L_m \to 0$, i.e.,

$$F_0 \approx -\frac{2\epsilon_0 V_g^2}{r_{\text{eff}}^2 \rho_v z \log^2(2z/r_0)}, \tag{26}$$

$$F_1 \approx \frac{4\epsilon_0 V_g^2}{\pi r_{\text{eff}}^2 \rho_v z^2 \log^2(2z/r_0)}, \tag{27}$$

$$F_2 \approx -\frac{\epsilon_0 V_g^2}{r_{\text{eff}}^2 \rho_v z^3 \log^2(2z/r_0)}, \tag{28}$$

$$F_3 \approx \frac{8\epsilon_0 V_g^2}{3\pi r_{\text{eff}}^2 \rho_v z^4 \log^2(2z/r_0)}, \tag{29}$$

where r_{eff} is the effective shell radius which takes into account the total mass average of the suspended beam. The static contribution of the external force will modify the equilibrium position of the beam, hence, induces residual tension along the tube. The first and third terms (F_1 and F_3) will modify the linear resonance frequencies and the non-linear behavior, respectively. The overall dynamic equation can be written as follows:

$$\ddot{u}_t + (\omega_0^2 - F_1)u_t + (\frac{E\pi^4}{2\rho_v L^4} - F_3)u_t^3 = F_0 + F_2 u_t^2 + \frac{F_{ac}}{m}, \tag{30}$$

or

$$\ddot{u}_t + (2\gamma + \gamma_3 u_t^2)\dot{u}_t + \omega_0^2(1-\beta)u_t + \alpha u_t^3 = Constant + \frac{F_{ac}}{m}, \tag{31}$$

where $\beta = F_1/\omega_0^2$, $\alpha = (E\pi^4)/(2\rho_v L^4) - F_3$, and the linear and nonlinear dissipation terms were included[19].

The solution for Supplementary Eq. 31 is found using the rotating frame approximation[18]. Briefly, one assumes external excitation of the form $\exp(i\omega_p t)$ and tries the following ansatz $u_t = (a - a^*)/(\Gamma - \Gamma^*)$, where $a = \mathbf{A}\exp(i\omega_p t)$, and $\Gamma = -\gamma + i\sqrt{\tilde{\omega_0}^2 - \gamma^2}$. In this notation we included the shift of the resonance frequency in $\tilde{\omega_0}$, i.e., $\tilde{\omega_0}^2 {=} \omega_0^2(1-\beta)$. Plugging this

approximation into the equation of motion and keeping only terms which change slowly with respect to ω_p results with the following implicit equation for $\mathbf{A}$

$$\mathbf{A} = \frac{F_{ac}}{2m} \frac{1}{i(\omega_p - \sqrt{\tilde{\omega_0}^2 - \gamma^2} - \kappa|\mathbf{A}|^2) + \gamma + \beta_3|\mathbf{A}|}, \tag{32}$$

where $\kappa = 3\alpha/8/(\tilde{\omega_0}^2 - \gamma^2)^{3/2}$, and $\beta_3 = \gamma_3\omega_p/8/(\tilde{\omega_0}^2 - \gamma^2)^{3/2}$. The solution for $|\mathbf{A}|$ is found from the following non linear equation

$$\left((\omega_p - \sqrt{\tilde{\omega_0}^2 - \gamma^2} - \kappa|\mathbf{A}|^2)^2 + (\gamma + \beta_3|\mathbf{A}|)^2\right) = \frac{1}{4}\frac{|F_{ac}|^2}{m}, \tag{33}$$

and the final temporal dependence of the tube reads the following

$$\begin{aligned} u_t(t) &= \frac{F_{ac}}{2m\sqrt{\tilde{\omega_0}^2 - \gamma^2}} \frac{\cos(\omega_p t - \phi)}{\sqrt{(\omega_p - \sqrt{\tilde{\omega_0}^2 - \gamma^2} - \kappa|\mathbf{A}|^2)^2 + (\gamma + \beta_3|\mathbf{A}|)^2}}, \\ \tan(\phi) &= \frac{\gamma + \beta_3|\mathbf{A}|)^2}{\sqrt{(\omega_p - \sqrt{\tilde{\omega_0}^2 - \gamma^2} - \kappa|\mathbf{A}|^2)^2 + (\gamma + \beta_3|\mathbf{A}|)^2}}. \end{aligned} \tag{34}$$

The linear and non-linear behaviors of the tube displacement were fitted to the maximal displacement of Supplementary Eq. 34 ($\cos = \pm 1$) self consistently with Supplementary Eq. 33. Examples for this fitted curves are depicted in Supplementary Figs. 22, 23, and Fig. 6. It is worth to mention that the nonlinear dissipation term, γ_3 is important for achieving better agreement between the experimental data and the theoretical fit, as depicted in Supplementary Figs. 22c, f.

The transition from hardening to softening, as depicted in Fig. 6, can be calculated within the Duffing lumped model. At low pressure according to our fitting (Fig. 6d), the nonlinear term α is found to be $\alpha = -0.56 \pm 0.07\, 10^{20}\,\mathrm{s^{-2}m^{-2}}$. According to our model both the

frequency and the nonlinear spring constant are affected by the electrostatic force (Supplementary Eq. 30). Both terms depend on several parameters such as slack, probe-tube distance, and tube length, all of which can be extracted from the optical image. The only two fitting parameters are r_1 and r_2, which are found from the dependance of the resonance mode on the external dc voltages at low excitation amplitude using our FEM (similar data as depicted in Supplementary Fig. 24). All together, we obtained from the Duffing lumped model $\alpha = -0.45 \pm 0.1\, 10^{20}\, \mathrm{s}^{-2}\mathrm{m}^{-2}$, where the error originates mainly from our uncertainty in the tube-probe distance, z. This estimated α is in a good agreement with the result of Fig. 6f.

Supplementary Note 8 Image processing

Dark field optical microscopy images of CNTs decorated with pNBA NCs form bright lines on a dark background. Using image processing techniques to detect nanotubes in those images is challenging due to the large amount of granular noise and the fact that nanotubes lines are usually faint and noncontinuous. For this aim, we use a two-stage approach. First, a Canny edge detector[20] is used to extract edges that form a rough estimation of the nanotubes layout. The Canny edge detector was selected due to its ability to detect a wide range of edges in the images. Then, we apply a set of post-processing techniques to the binary image of edges in order to remove noise and detect continuous curves. A morphological closing operation[21] is applied in order to fill gaps at the edges. The structuring element used for morphological closing is relatively small so that irrelevant elements situated close to a nanotube will not be merged with the nanotube curve. Then, connected component analysis[22] is used to remove these irrelevant elements. Different properties, such as the area and bounding ellipse, are calculated for each connected component. Nanotubes (and parts of nanotubes) can be distinguished due to their long and narrow bounding ellipse, and due

to their large area compared with irrelevant isolated elements. After imposing threshold for these properties, the remaining components consist mainly of disconnected nanotube parts and large irrelevant elements that are not close to the nanotubes. This allows for another iteration of morphological closing and threshold screening, this time with a larger structuring element that connects the nanotube parts, and higher thresholds that remove the remaining irrelevant elements. In the resulting binary image, pixels belonging to a nanotube are white and all other pixels are black. With this mask it is possible to mark the nanotubes in the original image and identify the starting and ending points of each nanotube, as shown in Fig. 7a.

After applying the image processing analysis to the optical images, a complete map of the CNTs location is found. Then, a layout of the circuit design is automatically obtained by imposing several design rules, such as electrodes separation, pads size, and single tube in the junction. An example of this process is presented in Supplementary Fig. 27, where inverter based on p and n type CNTFETs was planned. Supplementary Fig. 27a presents the CNT layout and the inverter design, all automatically obtained by our homemade computer code. The process includes three steps of e-beam lithography. In the first step strips for oxygen plasma were defined in order to remove the unnecessary tubes. In the second step electrodes for the two CNTs were fabricated. Next, we deposit 100 nm of SiO_2 on top of one CNT which suppose to remain p type at the end of the process. Than, a thin layer of HfO_2 was deposited all over the sample, and was etched from the device pads and from the area with the deposited SiO_2. Last, a quick wet etching in buffer oxide etch (BOE) removed the protected SiO_2 layer and rendered the device back again to be p-type FET. Supplementary Fig. 27b depicts the resulted inverter device that was design in Supplementary Fig. 27b. Alternative approach was based on two different metals for the p and n type

CNTFETs. The first step includes design of p-type CNTFET based on gold electrodes. The second step performs n-type CNTFET based on aluminum electrodes. The last step was designed for removing all other tubes that bridged the two junctions beside the desired one by rapid oxygen plasma. Supplementary Fig. 27c depicts the resulted behavior of a typical inverter that we have processed utilizing the automatic image processing algorithm.

Supplementary References

---

[1] Kong, J., Soh, H. T., Cassell, A. M., Quate, C. F. & Dai, H. J. Synthesis of individual single-walled carbon nanotubes on patterned silicon wafers. *Nature* **395**, 878–881 (1998).

[2] Ismach, A., Segev, L., Wachtel, E. & Joselevich, E. Atomic-step-templated formation of single wall carbon nanotube patterns. *Angew. Chem. int. Edit.* **43**, 6140–6143 (2004).

[3] Cao, J., Wang, Q. & Dai, H. Electron transport in very clean, as-grown suspended carbon nanotubes. *Nat. Mater.* **4**, 745–749 (2005).

[4] Pascal-Levy, Y. *et al.* Water-assisted mobile charge induced screening and origin of hysteresis in carbon nanotube field-effect transistors. *Phy. Rev. B* **86**, 115444(1–9) (2012).

[5] Javey, A., Wang, Q., Kim, W. & Dai. Advancements in complementary carbon nanotube field-effect transistors. *Int. Electron Devices Meeting Tech. Dig.* 741–744. (2003).

[6] Nosho, Y., Ohno, Y., Kishimoto, S. & Mizutani, T. n-type carbon nanotube field-effect transistors fabricated by using ca contact electrodes. *Appl. Phys. Lett.* **86**, 073105 (2005).

[7] Zhang, Z. *et al.* Doping-free fabrication of carbon nanotube based ballistic cmos devices and circuits. *Nano Letters* **7**, 3603–3607 (2007).

[8] Moriyama, N., Ohno, Y., Kitamura, T., Kishimoto, S. & Mizutani, T. Change in carrier type in high-k gate carbon nanotube field-effect transistors by interface fixed charges. *Nanotechnology* **21**, 165201 (2010).

[9] Blake, P. *et al.* Making graphene visible. *Applied Physics Letters* **91**, 063124 (2007).

[10] Smbles, J. R., Skinner, L. M. & Lisgarte, N. D. An electron microscope study of evaporating small particles - kelvin equation for liquid lead and mean surface energy of solid silver. *Proceedings of the Royal Society of London Series A-mathematical and Physical Sciences* **318**, 507–522 (1970).

[11] Jiang, Q. & Wen, Z. *Thermodynamics of Materials* (Springer, 2010).

[12] Landau, L. D. & Lifshitz, E. M. *Elasticity Theory* (Pergamon, Oxford, 1986., 1986).

[13] Ustunel, H., Roundy, D. & Arias, T. A. Modeling a suspended nanotube oscillator. *Nano Letters* **5**, 523–526 (2005).

[14] Krauthammer, E. V. T. *Thin Plates and Shells: Theory, Analysis, and Applications* (Marcel Dekker, Inc., 2001).

[15] Volterra, J., E. Morell. Lowest natural frequency of elastic arc for vibrations outside the plane of initial curvature. *Journal of Applied Mechanics* **28**, 624–627 (1961).

[16] Zienkiewicz, R., O.C. Taylor. *The Finite Element Method: Solid mechanics* (Butterworth-Heinemann, 2000).

[17] Witkamp, B., Poot, M. & van der Zant, H. S. J. Bending-mode vibration of a suspended nanotube resonator. *Nano Letters* **6**, 2904–2908 (2006).

[18] Nayfeh, A. *The Method of Normal Forms* (Wiley-VCH Verlag GmbH & Co. KGaA, 2011).

[19] Eichler, A. *et al.* Nonlinear damping in mechanical resonators made from carbon nanotubes and graphene. *Nature Nanotechnology* **6**, 339–342 (2011).

[20] Canny, J. A computational approach to edge detection. *IEEE transactions on Pattern Analysis and Machine Intelligence* **6**, 679 (1986).

[21] Soille, P. *Morphological closing operation* (Springer-Verlag New York, Inc., 2003).

[22] Gonzalez, R. C. & Woods, R. E. *Digital image processing* (2002).
